# Epigenetic Small Molecules Rescue Nucleocytoplasmic Transport and DNA Damage Phenotypes in C9ORF72 ALS/FTD

**DOI:** 10.3390/brainsci11111543

**Published:** 2021-11-20

**Authors:** Melina Ramic, Nadja S. Andrade, Matthew J. Rybin, Rustam Esanov, Claes Wahlestedt, Michael Benatar, Zane Zeier

**Affiliations:** 1 Center for Therapeutic Innovation, Department of Psychiatry & Behavioral Sciences, University of Miami Miller School of Medicine, 1501 NW 10th Ave, Miami, FL 33136, USA; mxr1727@miami.edu (M.R.); nadja.andrade@gmail.com (N.S.A.); mxr2011@miami.edu (M.J.R.); rustam.x.esanov@gsk.com (R.E.); cwahlestedt@med.miami.edu (C.W.); 2 Department of Neurology, University of Miami Miller School of Medicine, 1120 NW 14th St., Miami, FL 33136, USA; mbenatar@med.miami.edu

**Keywords:** ALS, C9ORF72, nucleocytoplasmic transport, RAN proteins, PR, GR, GA, arginine-rich peptides, dipeptide repeat proteins, iPSC motor neurons, high content screen, DNA damage

## Abstract

Amyotrophic lateral sclerosis (ALS) is a progressive and fatal neurodegenerative disease with available treatments only marginally slowing progression or improving survival. A hexanucleotide repeat expansion mutation in the *C9ORF72* gene is the most commonly known genetic cause of both sporadic and familial cases of ALS and frontotemporal dementia (FTD). The *C9ORF72* expansion mutation produces five dipeptide repeat proteins (DPRs), and while the mechanistic determinants of DPR-mediated neurotoxicity remain incompletely understood, evidence suggests that disruption of nucleocytoplasmic transport and increased DNA damage contributes to pathology. Therefore, characterizing these disturbances and determining the relative contribution of different DPRs is needed to facilitate the development of novel therapeutics for C9ALS/FTD. To this end, we generated a series of nucleocytoplasmic transport “biosensors”, composed of the green fluorescent protein (GFP), fused to different classes of nuclear localization signals (NLSs) and nuclear export signals (NESs). Using these biosensors in conjunction with automated microscopy, we investigated the role of the three most neurotoxic DPRs (PR, GR, and GA) on seven nuclear import and two export pathways. In addition to other DPRs, we found that PR had pronounced inhibitory effects on the classical nuclear export pathway and several nuclear import pathways. To identify compounds capable of counteracting the effects of PR on nucleocytoplasmic transport, we developed a nucleocytoplasmic transport assay and screened several commercially available compound libraries, totaling 2714 compounds. In addition to restoring nucleocytoplasmic transport efficiencies, hits from the screen also counteract the cytotoxic effects of PR. Selected hits were subsequently tested for their ability to rescue another C9ALS/FTD phenotype—persistent DNA double strand breakage. Overall, we found that DPRs disrupt multiple nucleocytoplasmic transport pathways and we identified small molecules that counteract these effects—resulting in increased viability of PR-expressing cells and decreased DNA damage markers in patient-derived motor neurons. Several HDAC inhibitors were validated as hits, supporting previous studies that show that HDAC inhibitors confer therapeutic effects in neurodegenerative models.

## 1. Introduction

Amyotrophic lateral sclerosis (ALS) remains a largely untreatable disease and, while rates of progression vary, patients typically have a life expectancy of about 2–5 years after symptom onset [1]. Currently, the only FDA-approved drugs for ALS are Riluzole and Edaravone [2,3]. Riluzole (the most commonly prescribed drug) prolongs survival by only 2–3 months [2], and Edaravone modestly slows the rate of functional decline [3]. There is, therefore, an urgent and unmet clinical need for more effective therapies to treat ALS patients.

A hexanucleotide (G_4_C_2_) repeat expansion (HRE) mutation in the *C9ORF72* gene is the most common known genetic cause of ALS and frontotemporal dementia (FTD), which is a related neurodegenerative disease [4,5,6,7]. Healthy individuals typically have less than 10 hexanucleotide repeats and may possess up to 30 repeats without significant risk of disease. Affected individuals, on the other hand, harbor large HREs composed of hundreds to thousands of repeats. Expansion length may vary among family members and across different tissues and brain regions of affected individuals, but the effects of repeat size on clinical phenotype, age of disease onset, and disease progression remains controversial [7,8,9,10,11,12,13]. The HRE leads to partial transcriptional repression of the *C9ORF72* gene, mediated through epigenetic disturbances and differential transcriptional start site utilization [14,15]. Despite the altered epigenetic status of expanded *C9ORF72* alleles, bidirectional transcription through the HRE produces mutant sense and antisense HRE RNAs. The high GC content of mutant RNAs thermodynamically favors the formation of stable G-quadruplex structures that facilitate translation of the HRE sequence, through a non-canonical mechanism called repeat-associated non-ATG (RAN) translation [16,17]. Differential reading frame utilization generates five different dipeptide repeat proteins (DPRs): Proline-Alanine (PA), Glycine-Alanine (GA), Glycine-Proline (GP), Glycine-Arginine (GR), and Proline-Arginine (PR). Among the DPRs, PR, GR, and GA are shown to induce length-dependent and dosage-dependent cytotoxicity in multiple model systems [18,19,20,21,22,23,24], with the arginine-rich DPRs, PR and GR, considered to be particularly deleterious. Despite substantial empirical evidence of DPR-mediated cytotoxicity, the underlying mechanisms remain incompletely defined and the contribution of DPRs to pathology is still debated.

It has been proposed that arginine-containing DPRs are potently cytotoxic, in part, due to their sub-cellular localization [25]. Since arginine-rich motifs of proteins may facilitate nuclear localization [26,27,28], PR and GR readily localize within the nucleus and nucleolus where they cause nuclear/nucleolar stress, impede DNA damage repair, and disrupt nucleocytoplasmic transport [22,29,30,31,32,33,34]. Functional genomic studies carried out by multiple independent groups produced strikingly similar results, whereby modulating the expression of genes encoding nucleocytoplasmic transport machineries was associated with the manifestation of phenotypes in yeast and *Drosophila* models of C9ALS/FTD [35,36,37]. Similarly, aberrant expression or localization of nucleocytoplasmic transport proteins has also been demonstrated in *C9ORF72*-induced, pluripotent stem cell (iPSC)-derived motor neurons [33,38]. These results, from multiple model systems, suggest that nucleocytoplasmic transport defects play an important role in *C9ORF72*–ALS/FTD pathology. However, most previous studies have only investigated classical nucleocytoplasmic transport pathways when, in-fact, a large family of karyopherins facilitate the import of cargo proteins via recognition of various classes of nuclear localization signals (NLSs). Moreover, previous studies have largely utilized model systems based on lower organisms and have not evaluated nucleocytoplasmic transport defects in mammalian cells. Here, we developed a systematic, high-throughput system to evaluate the effects of DPRs on both classical and non-classical nucleocytoplasmic transport pathways in human cells.

A number of NLSs have been functionally validated and can be transferred to unrelated proteins, such as GFP, to mediate nuclear localization [39,40,41]. Using this approach, we generated a battery of fluorescent nucleocytoplasmic transport “biosensors” by attaching various NLSs to GFP, allowing us to selectively probe specific nuclear import pathways. When expressed in mammalian cells, the subcellular localization of biosensors provides a direct fluorescent readout of transport dynamics, allowing for the assessment of nucleocytoplasmic transport perturbations. Using these pathway-specific GFP biosensors and a collection of several compound libraries, we aimed to measure the effects of toxic DPRs on classical and non-classical nucleocytoplasmic transport pathways and identify potential therapeutic compounds that restore disrupted nucleocytoplasmic transport in DPR-expressing mammalian cells.

Our previous work, and that of others, has established persistent DNA damage as another consequence of DPR expression that contributes to neurodegeneration in C9ALS/FTD [20,24,30,31]. We found that DPRs impede DNA double-strand break (DSB) repair pathways, leading to constitutive activation of repair machineries that can be detected by immunostaining with antibodies against various DSB markers, including γH2AX. In patient-derived iPSC motor neurons, γH2AX’s immunoreactivity is markedly increased as compared with genetically corrected motor neurons [30]. Using this robust cellular phenotype, we tested whether small molecules that restore nucleocytoplasmic transport defects are capable of rescuing DNA damage phenotypes as well.

## 2. Materials and Methods

### 2.1. Generation and Maintenance of Cell Lines with Constitutive Expression of Biosensors

To streamline the analysis of nucleocytoplasmic transport, enable screening assays, and to generate homogeneous population of cells with approximately equivalent levels of biosensor expression, U-2 OS cell lines that stably express each biosensor were generated. Briefly, U-2 OS cells (purchased from the American Type Culture Collection) were seeded into 6-well plates (300,000 cells per well). The following day, cells were treated with 4 μL of Lipofectamine 2000 CD (ThermoFisher, Waltham, MA, USA) and 3 μg of mammalian expression plasmids encoding biosensors and antibiotic resistance in serum-free media (OptiMEM; ThermoFisher). Cell cultures were allowed to recover for 24 h and GFP fluorescence was confirmed by UV microscopy. After 48 h, cells were subjected to selection using Geneticin (ThermoFisher) at 700 μg/mL for 7–14 days. Cells were propagated for at least 3 weeks and then sorted at least twice by fluorescence activated cell sorting (FACS). Sorting was performed to isolate cells with high GFP emission signal intensity to maximize biosensor detection in downstream analyses. All modified U-2 OS cell lines were maintained in DMEM with high glucose and L-glutamine (ThermoFisher), supplemented with 10% fetal bovine serum (FBS; ThermoFisher) and Geneticin (350 μg/mL), to maintain selection.

### 2.2. RNAi Transfection of U-2 OS Cells

U-2 OS cells were transfected with non-targeting negative control siRNA (Ambion, Austin, TX, USA) or siRNA directed against THOC4/ALYREF (Ambion, s19853) according to the manufacturer’s transfection protocol, using Lipofectamine RNAiMAX (ThermoFisher) as a transfection reagent and 25 pmol/L of siRNA per well in 6-well tissue culture plates.

### 2.3. DPR Transfection of U-2 OS Cells

To introduce DPRs, modified U-2 OS cells with constitutive expression of nucleocytoplasmic transport biosensors were transiently transfected with synthetic DPR expression plasmids, based on the pcDNA3.1+ backbone (described previously [30]). Briefly, 10,000 cells were seeded per well in a 96-well tissue culture plates. The following day, cells were transfected with 200 ng of PR_50_, GR_50_, and GA_50_ expression plasmids or the pcDNA3.1+ empty control vector using Lipofectamine 2000 CD (Invitrogen, Waltham, MA, USA), according to the manufacturer’s protocol. After 24 h, the cells were fixed and prepared for analysis by fluorescence microscopy.

### 2.4. iPSC Culturing and Motor Neuron Differentiation

iPSCs with the *C9ORF72* repeat expansion were created in the Zeier laboratory as previously reported (C9ALS-1, Isogenic-1) [14], or were purchased from the Cedars-Sinai iPSC core facility (https://biomanufacturing.cedars-sinai.org/, accessed on 18 October 2018; C9ALS-2, CS29iALS-nxx; C9ALS-3, CS52iALS-C9nxx; Isogenic-2, CS29iALS-C9n1.ISOxx; Isogenic-3, CS52iALS-C9n6.ISOxx). All iPSCs were propagated and used to generate neuronal cultures as previously described [14]. Briefly, iPSCs were seeded into Matrigel-coated flasks (Corning, 354277) and supplemented with mTeSR1 Plus medium (STEMCELL Technologies, Vancouver, BC, Canada, 85850). Once cultures reached ~80% confluency, cells were passaged using Gentle Cell Dissociation Reagent (STEMCELL Technologies, 07174) containing neuronal precursor media, consisting of neurobasal medium (Life Technologies, Carlsbad, CA, USA, 21103-049) supplemented with 2% NeuroCult SM1 (STEMCELL Technologies, 05711), 100 ng/µL human fibroblast growth factor (bFGF; PeproTech, 100-18B), 100 ng/µL epidermal growth factor (EGF; PeproTech, East Windsor, NJ, USA, AF-100-15), and 5 µg/µL heparin (Sigma-Aldrich, St. Louis, MO, USA, H3149-25KU). After 2 weeks of expansion and multiple passages, neuronal precursor cells were transferred to flasks coated in poly-L-ornithine (Sigma-Aldrich, A-004-M) or laminin (ThermoFisher, 23017015) and cultured in neuronal maturation media, consisting of DMEM/F12 (ThermoFisher, 11320033), supplemented with 1% N2 (Gibco, Waltham, MA, USA, A13707-01), 2% NeuroCult SM1 (STEMCELL Technologies, 05711), 1% Non-essential amino acids (Gibco, 11140-050), 2 µg/mL heparin (Sigma-Aldrich, H3149-25KU), 1% antibiotic/antimycotic (ThermoFisher, 15240112), 0.1 µM Retinoic acid (Sigma-Aldrich, 302-79-4), 1 µM purmorphamine (Sigma-Aldrich, 483367-10-8), 1 µM cAMP (Sigma-Aldrich, 60-92-4), 200 ng/mL ascorbic acid (Sigma-Aldrich, 50-81-7), 10 ng/mL glia-derived neurotrophic factor (GDNF; PeproTech, 450-10), and 10 ng/mL brain-derived neurotrophic factor (BDNF; PeproTech, 450-02). Neurons were cultured for at least 36 days in maturation medium, prior to experimentation. Efficiency of motor neuron differentiation was confirmed by immuno-staining for beta-tubulin III (TUJ1) (a general neuronal marker) and the insulin gene enhancer protein ISL-1 (a motor neuron specific marker), using the immunocytochemistry methods indicated below (see Figure S1).

### 2.5. Immunocytochemistry

For immunofluorescent visualization of U-2 OS and neuronal cell cultures, cells were fixed by exposure to 4% paraformaldehyde for 10 min, permeabilized for 15 min in 0.2% Triton X (Sigma-Aldrich) in PBS, and then blocked for 40 min in 0.2% Triton X and 20% goat serum (ThermoFisher) in antibody buffer. Cells were gently washed and treated with primary antibodies overnight at 4 °C (see Table S1 for a full list of antibodies and dilutions used). The next day, cells were washed 3 times with PBS, probed with secondary antibodies for 2 h, washed in PBS, and stained with DAPI to visualize cellular nuclei (ThermoFisher). U-2 OS cell cultures were imaged using the Cellomics VTI scanner for high content analysis. Immuno-labeled U-2 OS and neuronal cells were visualized using a Zeiss LSM 710 confocal microscope with 8-bit depth, 1024 × 1024 frame size; pixel intensity values were averaged 16 times at 20× or 63× magnification.

### 2.6. Semi-Automated Image Analysis

Confocal images were analyzed using ImageJ/FIJI. Object identification was performed by converting the DAPI emission channel to binary and analyzing particles to create an image mask representing cellular nuclei. Average nuclear intensity measurements were set to redirect to the γH2AX channel, measuring the average γH2AX fluorescence intensity within the nucleus of each cell. To reduce artifact signal, outliers were removed from the datasets using a MATLAB script, whereby data points that fell below the inner quartile × 1.5 the interquartile range, or fell above the outer quartile × 1.5 the interquartile range, were considered outliers.

### 2.7. Automated Microscopy and Image Analysis

All automated image acquisition and analysis activities were performed using the Cellomics Arrayscan VTi automated imaging system in collaboration with the High Content Screening core facility at the University of Miami Miller School of Medicine. A fluorescent dye, DAPI, was used to stain DNA and distinguish the nuclear–cytoplasmic boundary. During image acquisition, the DAPI emission signal was used for instrument autofocusing. Sequential acquisition of images yielded separate files for DAPI and GFP emission signals for each field. Following image acquisition, algorithms for pattern recognition within the ArrayScan VTi software was used to compute an image mask, separating the signal from background. Image files overlaid with the image mask were visually inspected to assess the performance of nuclei or “object” recognition. Manual background correction and threshold settings were used to optimize object segmentation. Image segmentation based on differential DAPI emission signal intensity defined the nuclear–cytoplasmic boundary mask, whereby a circle area (nucleus) was computed for each valid object (cell). Then, a ring area, spatially located around each nuclei trace, was computed to represent the cytosol. After defining sub-cellular compartments with image masks, the circle–ring GFP signal intensity difference was computed as a population average across valid objects within each well. We termed the circle–ring GFP intensity difference the “translocation index” of biosensors, and this parameter was used as the primary assay read-out.

### 2.8. High Content Image Data Analysis

To distinguish between dead or apoptotic cells with condensed chromatin and viable cells with larger nuclei, we analyzed the DAPI image mask to identify circles (nuclei) with high signal intensity, small sizes, and aberrant shapes. Nuclei that exceeded threshold settings were identified as “invalid objects”, representing dead or dying cells in each well. Invalid object counts were used to assess cellular viability and were excluded from downstream analysis of nucleocytoplasmic transport. Wells with “invalid objects” above 2 standard deviations of the plate mean were not considered during hit identification, due to the assumption of cellular toxicity. We optimized transfection efficiency of DPRs by calculating the number of cells expressing DPRs in each well, as determined by immunostaining. DPR positive cells were determined by setting thresholds for the intensity of immuno-labeled DPRs in the circle and ring of each cell. Wells with DPR positive cells bellow 2 standard deviations of the plate mean were not considered during hit identification, due to the assumption of insufficient DPR expression. Individual cells with average GFP intensities in the circle or ring, that exceeded 3 standard deviations above or below the mean, were considered outliers and were removed from the analysis. We calculated Z’ across control wells for each read-out to assess assay robustness [42]. Resulting data were analyzed for translocation index, the primary assay readout, and a Z’ value calculated for control wells. Plates with Z’ values less than 0.5 were removed from further analysis, as this is a standard benchmark for robust assays in high-throughput screening. The mean translocation index of each well was normalized to their in-plate control wells, then a Z-factor of the translocation index was calculated across all tested compounds. A rank order of 20 compounds with the most negative Z-factor and highest cell viability was used to identify hits.

### 2.9. Statistical Analysis

Figure legends provide information regarding experimental replications. Student’s *t* Test was used to determine statistical differences between two means. One-way or two-way ANOVA followed by Sidak’s multiple comparison or Dunnett’s multiple comparison test was used to determine statistical differences between two or more means. Data values represent mean ± SD. GraphPad Prism and MATLAB were used to perform statistical analyses.

The Z’ (Z-Factor) for every plate used for high content analysis was calculated using the following equation:

$$Z^{\prime }=1-\frac{3(\sigma p+\sigma n)}{\mu p-\mu n}$$

The variables refer to the means (*µ*) and standard deviations (*σ*) of the positive (*p*; leptomycin-b) and negative (*n*; DMSO) controls [42]. The Z-factor of the translocation index for every compound was calculated using the following equation:

$$Z=\frac{X-\mu }{\sigma }$$

The variables refer to the individual translocation index (*X*), the mean translocation index of all compounds (*µ*), and the standard deviation of all compounds (*σ*).

## 3. Results

### 3.1. Nucleocytoplasmic Transport Biosensors Are Functional in Mammalian Cells

**Figure 1:**
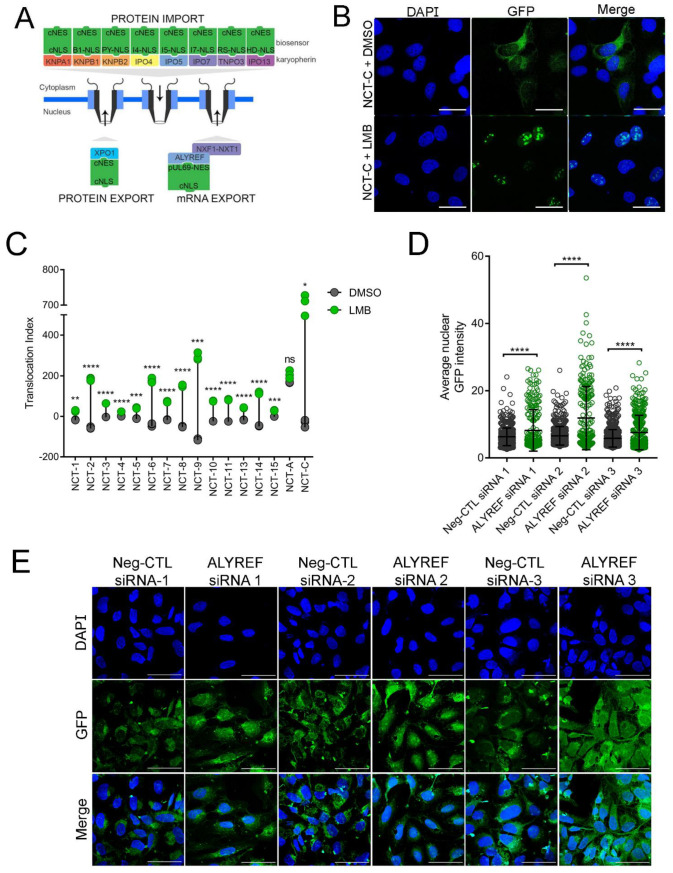
Nucleocytoplasmic transport biosensors are functional. (**A**) Schematic of biosensor design—a green fluorescent protein fused with different classes of nuclear localization signals (NLSs) and the classical nuclear export signal (cNES) or the Aly/REF recognition motif. (**B**) Representative confocal images of cells expressing the NCT-C biosensor (green) under control conditions (DMSO) and after exposure to leptomycin (LMB) (2 h at 2 ng/mL). Scale bar = 40 µm. (**C**) Dot plot of the LMB-induced translocation for each biosensor relative to vehicle. Each dot represents a technical replicate. (**D**) Quantification of average nuclear intensity of GFP using image analysis software ImageJ for three biological replicates of the NCT-A cells transfected with either negative control siRNA or siRNA targeting Aly/REF. Each data point represents one cell. (**E**) Representative confocal immunofluorescence images of NCT-A U-2 OS cells stained with DAPI. Scale bars = 40 µm; n = 3 biological replicates; 5 fields per replicate were imaged; error bars are SD. (Students *t*-test: * *p* < 0.05; ** *p* < 0.01; *** *p* < 0.001; **** *p* < 0.0001; ns: non-significant).

Multiple nucleocytoplasmic transport pathways mediate the movement of proteins and RNAs through the nuclear pore complex [43]. While there are substantial redundancies in the recognition of NLSs by importins, several classes of NLSs are selectively recognized by a particular importin [44]. By retrofitting GFP with these selectively recognized NLSs, we developed a method to probe distinct import pathways and investigate whether they are dysregulated in C9ORF72 ALS/FTD (Table 1 and Figure S2). To interrogate the classical import and export pathways, we utilized a previously described biosensor, pRevMAPKKnesGFP, which was a gift from Beric Henderson and Wolfgang Link [40]. Stable expression of the pRevMAPKKnesGFP biosensor was established in U-2 OS cells and the cell line was named NCT-C. The biosensor contains an arginine-rich NLS (^35^RQARRNRRRRWRERQRQ^51^), discovered in the HIV-1 Rev protein, that recognizes importin-β1 directly, as well as a nucleolar localization signal (NuLS). We also designed and generated our own biosensors utilizing the major classes of NLSs: bipartite cNLS (NCT-03), proline-tyrosine NLS (PY-NLS; NCT-07, NCT-08, and NCT-09), and arginine-serine NLS (RS-NLS; NCT-13; Figure 1A) [45,46,47,48]. In addition, several previously validated, but lesser-known NLSs were included to probe non-classical import pathways (NCT-04, NCT-05, NCT-06, NCT-10, NCT-11, and NCT-14) (Table 1) [49,50,51,52,53]. To confer biosensor export, we utilized the classical nuclear export signal (cNES), the only well-characterized NES for proteins [54,55,56]. For messenger RNA (mRNA), the prototypical export pathway requires the Aly/REF export factor protein, also known as THO complex subunit 4 (THOC4), encoded by the ALYREF gene. Aly/REF mediates mRNA export by interacting with the nuclear RNA export factor 1 (NXF1) and nuclear transport factor 2 (NTF2) related export protein 1 (NXT1) heterodimer [57,58]. Because mRNA processing is disrupted in C9ORF72 ALS/FTD [59,60,61,62], we explored this pathway as well as protein export by generating the mRNA export biosensor—NCT-A (Figure 1A and Table 1).

After generating mammalian expression vectors encoding each biosensor, stable expression was established by selection in U-2 OS cells, which are commonly utilized in image-based assays due to their advantageous morphology (large cells with easily distinguishable sub-cellular compartments). Due to the efficiency of the cNES, relative to NLSs, biosensors predominantly localize to the cytoplasm under baseline conditions, as can be seen in confocal images of NCT-C treated with vehicle (DMSO; Figure 1B). To confirm the functionality of biosensors, U-2 OS cells stably expressing each biosensor were exposed to leptomycin-b (LMB), a well-known inhibitor of XPO1 that binds covalently to a cysteine residue in a central conserved region of the cNES [63,64]. Using automated image acquisition and analysis software (see methods), we quantified the subcellular shift (translocation index) of our biosensors in response to LMB. Using the emission signal of DAPI we generated an image mask demarking the nucleus and cytoplasm. The difference between GFP emission signal intensity in the nucleus (circle) and cytoplasm (ring) was computed and this translocation index was used as the primary readout. Inhibition of XPO1 by exposure to LMB caused an accumulation of cNES containing biosensors in the nucleus, resulting in a positive translocation index value (Figure 1B,C), thereby confirming the functionality of the biosensors. Representative images can be found in supplementary Figure S3. As expected, the biosensor containing an NES, recognized by the Aly/REF export factor (NCT-A), does not accumulate in the nucleus in response to LMB, since export of NCT-A is not mediated through XPO1. The functionality of the NCT-A biosensor was validated by siRNA knockdown of Aly/REF, where an increase in average nuclear GFP intensity was observed after Aly/REF knockdown, but not cells transfected with a negative control siRNA (Figure 1D,E). Overall, these results support the functionality of our nucleocytoplasmic biosensors in mammalian cells.

### 3.2. Proline-Arginine Disrupts the Classical Nucleocytoplasmic Transport Pathway

**Figure 2:**
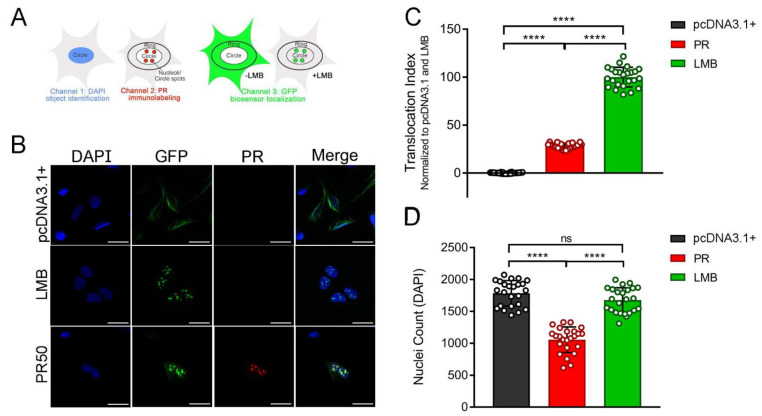
PR_50_ alters the cellular localization of the NCT-C biosensor. (**A**) Schematic of the NCT-C biosensor and automated image segmentation strategy. U-2 OS cells expressing the biosensor composed of GFP fused with the Rev-NLS and cNES (green). Nuclei were stained by exposure to DAPI (blue). Image acquisition corresponding to the emission wavelengths (channels) of DAPI, immunolabeled PR (red), and GFP was performed using a Cellomics Arrayscan VTI scanner. Image segmentation was carried out using the HCS Studio software that identifies differences in signal intensity for each channel. Pixels corresponding to cellular nuclei (circles) were assigned by high DAPI signal intensity while the surrounding image area was designated as cytoplasm (ring). (**B**) Confocal images of U-2 OS cells expressing the NCT-C biosensor (green) transfected with empty vector control (pcDNA3.1+), 2 h LMB treatment (2 ng/mL), or 24 h PR_50_ overexpression conditions. Scale bar = 40 µm. (**C**) Quantification of the NCT-C biosensor translocation index (circle average GFP intensity–ring average GFP intensity), normalized to pcDNA3.1+ (0%) and LMB (100%) in U-2 OS cells. Each data point represents a technical replicate. One-way ANOVA; F (2, 69) = 1683; **** *p* < 0.0001. (**D**) Quantification of cell counts in U-2 OS cells transfected with empty vector, treated with LMB (2 h at 2 ng/mL), or 24 h PR_50_ overexpression conditions. One-way ANOVA; F (2, 69) = 94.01; **** *p* < 0.0001; ns = non-significant.

Genetic screens have shown that modifiers of PR- and HRE-induced toxicity overwhelmingly cluster within nucleocytoplasmic transport and nuclear pore complex genes [35,36,37]. In order to assess the potential disruption of classical nucleocytoplasmic transport pathways in response to PR, we performed a high-throughput microscopy experiment using cells that stably express the NCT-C biosensor. At 24 h post-transfection with the PR_50_ expression vector, we used an automated image acquisition and analysis assay to evaluate the effects of PR_50_ on the subcellular localization of the NCT-C biosensor (Figure 2A). Using a statistical measure, Z-factor [42], we compared the effect size between the control wells: transfection with empty plasmid vector (pcDNA3.1+) and treated with either LMB or vehicle (DMSO). For all downstream experiments, we used a quality control threshold of Z-factor > 0.5 to authenticate biosensor functionality and assay robustness.

Transfection of the PR_50_ expression plasmid caused a robust translocation shift due to the accumulation of NCT-C in nuclei (Figure 2B,C)—a similar effect was observed after exposure to LMB (2 ng/mL for 2 h). Since LMB is an extremely efficient XPO1 inhibitor, we normalized the translocation index for each biosensor, whereby baseline conditions (pcDNA3.1+) were scaled to 0% translocation (primarily cytoplasmic) and LMB to 100% translocation (primarily nuclear). When quantified, PR_50_ caused a significant 30% increase in translocation index value compared with the baseline, corresponding to biosensor accumulation in the nucleus and nucleolus (Figure 2C). Additionally, wells transfected with the PR_50_ expression plasmid had significantly decreased cell counts compared with the empty vector control wells, consistent with the known cytotoxic effects of PR expression (Figure 2D). There was no significant change in cell counts between the control and LMB-treated wells, suggesting the concentration of LMB used in the assay was sufficient to block nuclear export but was not measurably cytotoxic at the time of cell fixation and analysis. In summary, overexpression of synthetic PR_50_ caused a disruption in nucleocytoplasmic transport and a decrease in cell viability, supporting the notion that PR is toxic and has a direct role in disrupting nucleocytoplasmic transport, as has been previously reported by others [18,21,30,65].

### 3.3. Poly-Dipeptide Repeat Proteins Disrupt the Nuclear Export of Proteins but Not mRNA

**Figure 3:**
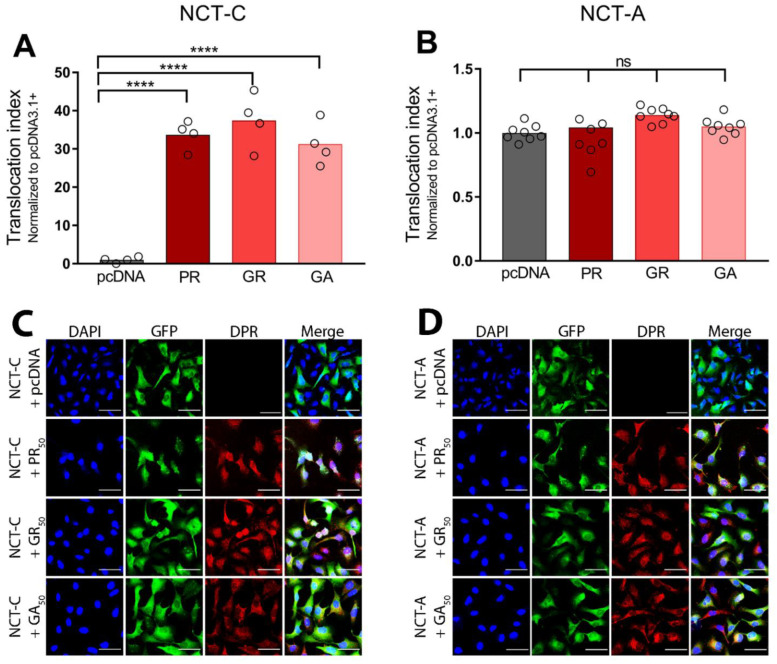
DPRs disrupt the classical nuclear export pathway. (**A**) Bar graph of the translocation index of the NCT-C biosensor after 24 h transfection of an empty vector control (pcDNA3.1+), PR_50_, GR_50_, or GA_50_. Translocation indices were normalized to pcDNA3.1+ (1 = GFP predominantly cytoplasmic). Each condition was performed with 4 technical replicates. (**B**) Bar graph of the translocation index of the NCT-A biosensor after 24 h transfection of an empty vector control (pcDNA3.1+), or PR_50_, GR_50_, or GA_50_. Translocation indices were normalized to pcDNA3.1+ (1 = GFP predominantly cytoplasmic). Each condition was performed with 8 technical replicates. (**C**) Representative images of U-2 OS cells expressing the NCT-C biosensor (green) transfected with empty vector control (pcDNA), or 24 h PR_50_, GR_50_, or GA_50_ overexpression conditions. Scale bar = 80 µm. (**D**) Representative images of U-2 OS cells expressing the NCT-A biosensor (green), transfected with empty vector control (pcDNA), or 24 h PR_50_, GR_50_, or GA_50_ overexpression conditions. Scale bar = 80 µm. Two-way ANOVA; F (3, 40) = 101.8; **** *p* < 0.0001; ns = non-significant. Statistical results can be found in Table S2.

To evaluate the potential contribution of other cytotoxic DPRs to nucleocytoplasmic transport deficiencies in C9ALS/FTD, we measured the effects of GR and GA on NCT-C translocation. Similar to PR, GR is cytotoxic and has been associated with compromised mitochondrial function, increased oxidative stress, DNA damage, disrupted protein translation, and impaired stress granule formation [21,24,30,37,66]. While generally less cytotoxic than arginine-rich DPRs, GA is the most abundant DPR and the most prone to forming cytoplasmic aggregates [20,67]. After 24 h of DPR overexpression in NCT-C cells, we found PR_50_ and GR_50_ localized predominantly in the nucleus, while GA_50_ was primarily cytoplasmic (Figure S4). Our results indicated that all three DPRs significantly increased the translocation index for the NCT-C biosensor when compared with the baseline (Figure 3A; see Table S2 for statistical results across all biosensors).

To investigate how nuclear export of macromolecules might be disrupted by DPRs, we quantified and compared the translocation index of the NCT-C and NCT-A biosensors in response to PR_50_, GR_50_, or GA_50_. While the NCT-C biosensor utilizes the cNES that is recognized by XPO1, the NCT-A biosensor contains a NES recognized by Aly/REF, the mechanism typically utilized for mRNA export. None of the DPRs affected the subcellular localization of the NCT-A biosensor, relative to the empty vector control (Figure 3B). These data suggest that DPRs disrupt classical protein export mediated by XPO1, but do not interfere with Aly/REF-mediated mRNA export.

### 3.4. Poly-Dipeptide Repeat Proteins Inhibit Multiple Nuclear Import Pathways

After investigating the classical import and export pathways, we next sought to determine whether other nuclear import pathways are also affected by the expression of DPRs. To this end, we generated several nucleocytoplasmic transport biosensors with different classes of NLSs paired with an identical cNES. Since these remaining biosensors utilize the same cNES, the potential effects of DPRs on nuclear export can be assumed to be equivalent for each biosensor; therefore, relative changes in the translocation index can be attributed to effects on nuclear import. As before, we normalized data according to each biosensor’s dynamic range by scaling the translocation index to baseline conditions (pcDNA3.1+) set to 0% and LMB exposure set to 100%.

**Figure 4:**
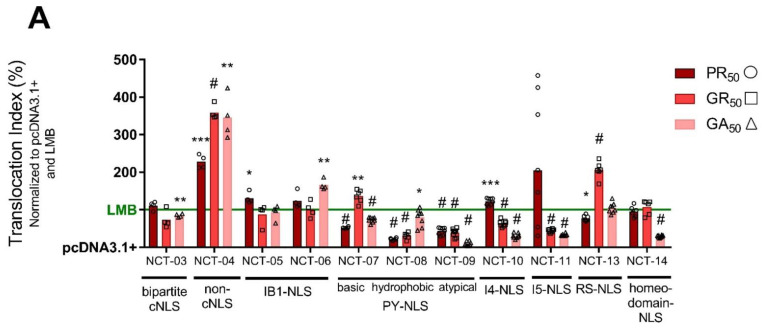
Dipeptide repeat proteins disrupt non-classical nuclear import pathways. Bar graph of the translocation index of each NCT-biosensor-expressing U-2 OS cell line under 24 h transfection of an empty vector control (pcDNA3.1+), PR_50_, GR_50_, or GA_50_ or 2 h LMB treatment (2 ng/mL). Data was normalized to pcDNA3.1+ (0%) and LMB (100%; green line). Each data point represents a single replicate. Each condition was performed with 4 to 8 technical replicates. Two-way ANOVA; asterisks or pound sign indicates significance, relative to LMB; F (20, 167) = 9.882; # *p* < 0.0001; *** *p* < 0.001; ** *p* < 0.01; * *p* < 0.05; no asterisks or pound sign = non-significant. Statistical results can be found in Table S2.

We found that GA expression caused significantly less nuclear accumulation of NCT-03 (the bipartite cNLS utilized by TDP-43) when compared with LMB (Figure 4). Previous evidence suggests that GA toxicity is due to the formation of cytoplasmic aggregates and sequestration of proteins [19,20,68]. Particularly, GA aggregates have been shown to prevent the nuclear import of TDP-43 and increase DNA damage [69]; therefore, our results support the idea that GA interferes with the import of proteins with a bipartite cNLS, such as TDP-43 [70,71]. Furthermore, PR_50_, GR_50_, and GA_50_ caused a significant increase in nuclear localization of NCT-04, a biosensor that employs a non-classical NLS that is less arginine-rich and, thus, less basic than the cNLS (Figure 4). There is evidence that arginine-rich DPRs disrupt import of cargo containing a cNLS motif by directly binding to importins and blocking the cNLS binding site [71]. These findings suggest that DPRs pose an inhibitory effect on the import of cargo containing the cNLS as opposed to a non-classical NLS whose interaction with importin-α only partially overlaps the cNLS binding site. Additionally, PY-NLS motifs, while much more diverse in amino acid sequence, also contain an overall positive charge and stretches of arginine-rich sequences like the cNLS [47]. This could explain why DPRs caused significantly lower nuclear GFP intensities compared with LMB in our NCT-07, NCT-08, and NCT-09 biosensors (Figure 4). Overall, PR_50_, GR_50_, and GA_50_ individually produce a wide array of nucleocytoplasmic transport disruptions across multiple NLS types.

### 3.5. Small Molecules Targeting Epigenetic Modifiers Restore Disrupted Nucleocytoplasmic Transport in PR_50_ Expressing U-2 OS Cells

**Figure 5:**
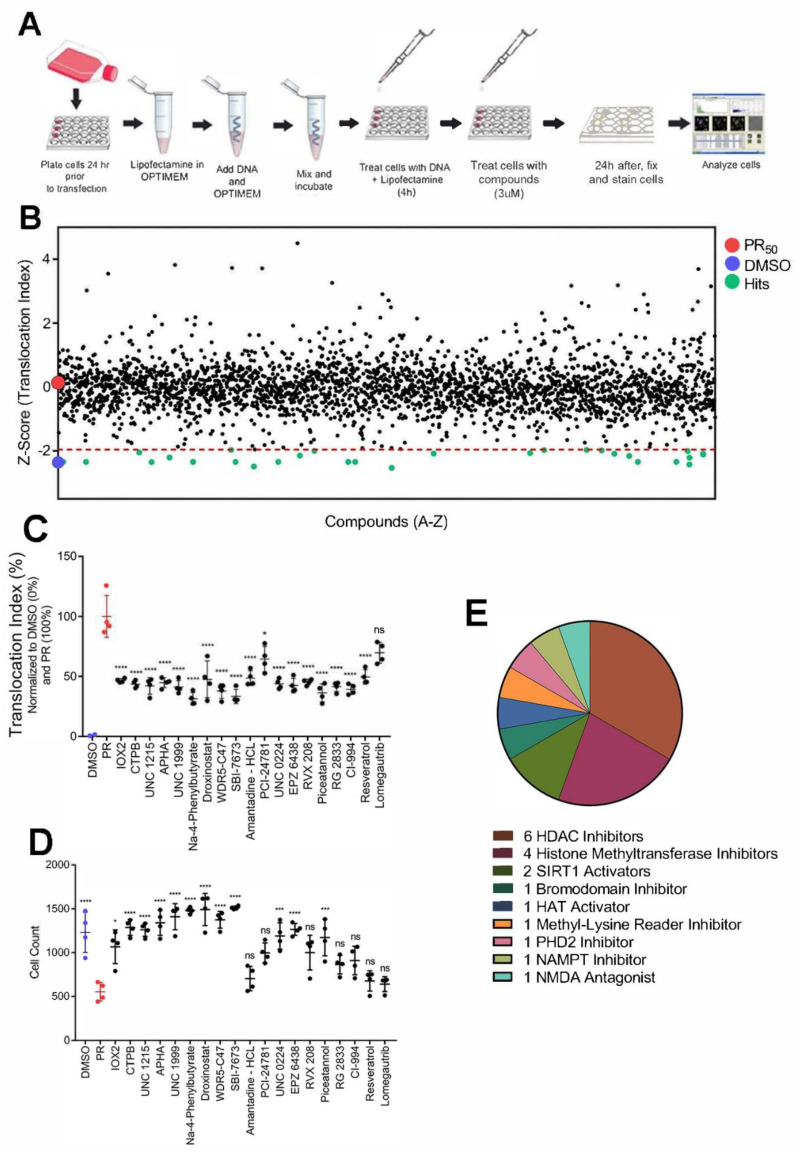
A phenotypic screen for modifiers of disrupted nucleocytoplasmic transport in U-2 OS cells expressing PR_50_. (**A**) Schematic of the phenotypic screen workflow. U-2 OS cells stably expressing NCT-C biosensor were plated 24 h prior to PR_50_ transfection and treated with compounds at 3 µM 4 h thereafter. After 24 h of compound exposure, plates were fixed with 4% paraformaldehyde, stained with DAPI and an anti-PR antibody prior to image acquisition. (**B**) Scatterplot of compound Z-factors across all compound libraries at a single concentration (3 µM) in the presence of PR_50_. A more negative Z-factor indicates restored NCT-C; whereas, a more positive Z-factor indicates disrupted NCT-C. (**C**) A confirmatory screen of the translocation index of top compounds from the primary screen mentioned above. Cells were either treated with vehicle (DMSO), untreated and expressing PR_50_ (PR), or treated with compound at 3 µM for 24 h in the presence of PR_50_. Each condition was repeated 4 times. (**D**) A scatterplot of cell counts under each condition from the confirmatory screen mentioned above. (**E**) A pie chart indicating the class of each hit compound tested in the confirmatory screen. * *p* < 0.05; *** *p* < 0.001; **** *p* < 0.0001; ns = non-significant.

We found that PR overexpression disrupts nucleocytoplasmic transport and decreases cell viability (Figure 2). Therefore, compounds that restore nucleocytoplasmic transport could mitigate the toxic effects of PR and inform new therapeutic strategies. To this end, we screened 2714 compounds, including FDA-approved compounds, an epigenetic-focused library, natural compounds, and other pharmacologically active compounds at 3 µM for 24 h, with translocation index of the NCT-C biosensor as the primary assay readout (Figure 5A; see Table S3 for details on compound libraries). Assay performance for each 96-well plate was determined by calculating the Z-factor between vehicle (DMSO) and LMB control wells (Figure S5). Plates not exceeding a Z-factor threshold of 0.5 were excluded from the study and replicated until the quality control standard was met. Normalization of translocation indices was performed by min–max scaling, using in-plate DMSO wells as the minimum value and PR control wells as the maximum value. After normalization to control wells, translocation index changes in the presence of PR and each compound were identified by calculating a Z-factor for each test well. The Z-factor is a statistical comparison between a given well and all other test wells within the plate (see methods for details of statistical tests utilized). Using this strategy, the primary screen identified 47 compounds with a Z-factor threshold of <−1.96, indicating reversal of PR-induced translocation of the NCT-C biosensor (Figure 5B). Several of the compounds were epigenetic modulators, particularly HDAC inhibitors (Table S4). Of the preliminary hits, we selected 18 compounds that also increased cell viability in PR_50_-expressing cells for further validation. A confirmatory screen with four technical replicates at the primary screen latency (24 h) and concentration (3 µM) was carried out (Figure 5C). All 18 hits were confirmed to counteract the effects of PR on nucleocytoplasmic transport. Thirteen of the 18 confirmed hits also significantly increased cell counts, as compared with PR-expressing cells (Figure 5D). Additionally, two compounds—Na-4-Phenylbutyrate and UNC 1999—reduced the nuclear intensity of PR, shifting it to the cytoplasm (Figure S6). To ensure the accuracy of our confirmatory screen, we included the MGMT (O6-methylguanine-DNA methyltransferase) inhibitor, lomegautrib, as a control that was not identified as a hit in the primary screen. As expected, lomegautrib did not significantly decrease the translocation index of the NCT-C biosensor or increase cell viability in PR_50_-expressing cells. Among the 13 hits, 6 were HDAC inhibitors and 4 were histone methyltransferase inhibitors (Figure 5E). These data suggest that small molecules targeting epigenetic proteins, primarily HDACs and histone methyltransferases, may have therapeutic potential through the restoration of disrupted nucleocytoplasmic transport.

### 3.6. Small Molecules Targeting Epigenetic Modifiers Reduce γH2AX Immunoreactivity in C9ALS/FTD Patient iPSC-Derived Motor Neurons

Our cell-based phenotypic assay identified small molecules that showed therapeutic potential by restoring PR-induced disruption of nucleocytoplasmic transport and increasing U-2 OS cell viability. However, validating these small molecules in a more disease-relevant model system, such as iPSC motor neurons (MNs), is necessary. Unlike U-2 OS cells, iPSC motor neurons are not morphologically amenable to the nucleocytoplasmic transport assay. Therefore, we sought to determine whether hits from our screen could rescue another PR-induced cellular phenotype. In a recent publication, we showed a robust DNA damage phenotype in our C9ALS MNs, whereby a significant increase in DNA DSBs can be observed in patient MNs relative to their isogenic controls as determined by immunostaining for DSB marker proteins like γH2AX [30]. Additionally, by using U-2 OS DNA DSB to repair pathway-specific reporter cell lines, we found that PR overexpression caused pronounced inhibitory effects on NHEJ (non-homologous end joining), MMEJ (micro-homology end joining), and SSA (single-strand annealing) repair pathways. We have previously generated iPSC MNs using one C9ALS/FTD iPSC line (C9ALS-1) from our local patient population [14]. In addition, we excised the *C9ORF72* expansion mutation and generated an isogenic control cell line via transduction with recombinant adeno-associated viral vectors expressing the Cas9 endonuclease and guide RNAs flanking the HRE (Isogenic-1) [30]. We obtained two additional patient iPSC cell lines from the Cedars-Sinai iPSC core facility and their corresponding isogenic controls (C9ALS-2 and Isogenic-2, C9ALS-3 and Isogenic-3). To confirm efficient neuronal differentiation, motor neuron cell cultures were stained for ISL-1—a motor neuron-specific marker (Figure S1).

Knowing this, we asked whether treating C9ALS MNs with the hit compounds from the nucleocytoplasmic transport screen would decrease overall γH2AX levels in C9ALS/FTD motor neurons. Through immunofluorescence and semi-automated imaging analysis with ImageJ, we first reproduced our previous results, showing that C9ALS MNs have significantly increased nuclear fluorescence intensity of γH2AX relative to their corresponding isogenic controls (Figure S7). A 1 h exposure to 200 μM etoposide to induce DSBs was used as a positive control. As expected, etoposide significantly increased the nuclear intensity of γH2AX immunoreactivity in all of the C9ALS/FTD lines and their isogenic controls.

We focused on hit compounds that counteracted the effects of PR on nucleocytoplasmic transport and conferred the greatest increase in U-2 OS cell viability. Among several hits that were HDAC inhibitors or EZH1/2 (enhancer of zest homologue 1 or 2, or both) methyltransferase inhibitors, we selected a brain-penetrant pan-HDAC inhibitor Na-4-phenylbutyrate, an EZH2-specific inhibitor EPZ-6438, the CBP/p300 histone acetyltransferase (HAT) activator CTPB, and an SIRT1 activator piceatannol. A recent study by Fazal et al. showed that HDAC6 inhibition specifically restores TDP-43 pathology and transport defects in iPSC motor neurons [72]. Therefore, to explore the role of HDAC6, we included an HDAC6-specific inhibitor CAY10603 as well as Quisinostat, a broad spectrum HDAC inhibitor that does not inhibit HDAC6. Appropriate doses for our iPSC motor neurons were determined by a 3-point dose response (1 × IC_50_, 10 × IC_50_, and 100 × IC_50_) in C9ALS-3 MNs (Figure S8). Doses that had a greater decrease in nuclear γH2AX levels were chosen for treatment in MNs derived from 3 separate C9ALS/FTD iPSC lines.

**Figure 6:**
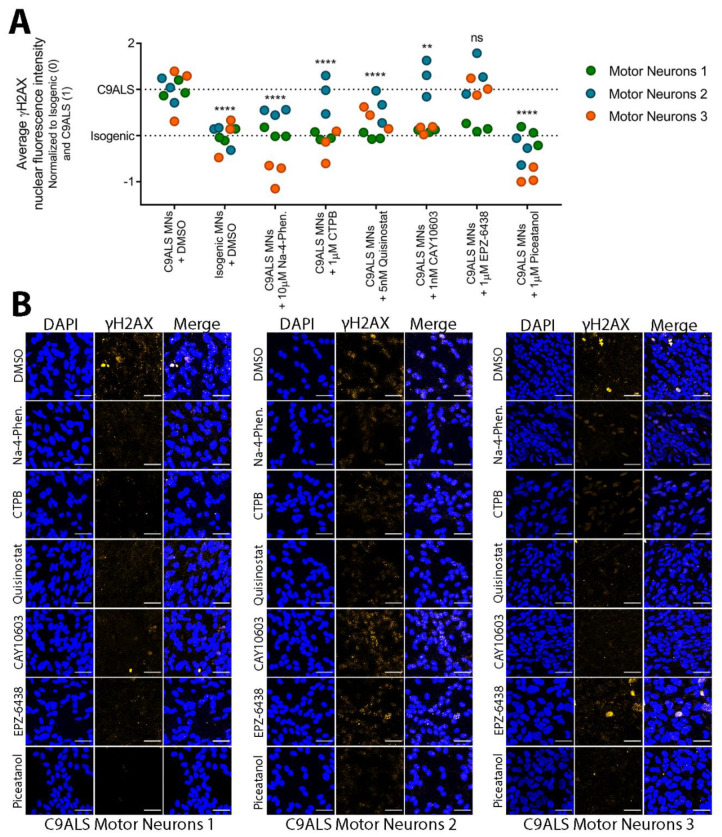
Small molecules targeting epigenetic modifiers decrease DNA damage marker γH2AX in C9ALS patient iPSC-derived motor neurons. (**A**) Quantification of average γH2AX nuclear fluorescence intensity in each C9ALS MN (C9ALS-1—green; C9ALS-2—blue; C9ALS-3—orange) or their isogenic controls treated with either vehicle (DMSO) or various doses of the small molecules. Data was normalized across all three C9ALS MNs by setting baseline (0%) to the Isogenic + DMSO γH2AX levels and setting maximum γH2AX nuclear intensity (100%) to the C9ALS + DMSO γH2AX levels. n = 3 biological replicates. There are 3 technical replicates per biological replicate. Each dot represents 1 technical replicate. Two-way ANOVA with multiple comparisons using Dunnett’s correction; F (7, 48) = 25.51; **** *p* < 0.0001; ** *p* < 0.01; ns = not significant. (**B**) Representative confocal images for data represented in (**A**), showing DAPI nuclei staining and γH2AX (orange). Scale bar = 40 μm. Semi-automated image analysis was carried out using confocal imaging and ImageJ. Statistical analysis performed in Graphpad Prism.

After a 72 h treatment with small molecules, cells were fixed and stained with a validated antibody against γH2AX. Overall, the HDAC inhibitors, HAT activator, and SIRT1 activator significantly decreased the average nuclear fluorescence intensity of γH2AX in C9ALS motor neurons (Figure 6A,B). The EZH2 inhibitor EPZ-6438 did not reduce γH2AX levels. While several of these compounds decreased DSBs when data from all three of our C9ALS iPSC MNs were analyzed together, it should be noted that variation was observed between each patient cell line. In particular, C9ALS-1 responded robustly to each treatment, including EPZ-6438, with nuclear γH2AX levels significantly decreased when analyzed separately from MNs derived from the other two iPSC lines (See Table S5 for detailed statistical results). C9ALS-2, however, only responded significantly to Na-4-phenylbutyrate and piceatannol treatment, while C9ALS-3 responded significantly to Na-4-phenylbutyrate, CTPB, EPZ-6438, and piceatannol treatment.

To further assess the impact of small molecules on NCT, we asked if any hit compounds could restore the localization of TDP-43, whose mis-localization to the cytoplasm is a hallmark of ALS [70,73,74,75]. To determine if our C9ALS iPSC MNs exhibit TDP-43 pathobiology, we first measured nuclear TDP-43 levels in our C9ALS iPSC MNs and their isogenic controls by immunofluorescence and ImageJ analysis (Figure S8). Only C9ALS-3 had significantly less nuclear TDP-43 compared with its isogenic control, suggesting that C9ALS-3 MNs manifest TDP-43 mis-localization. After treating C9ALS-3 MNs with each hit compound, however, none of the compounds significantly increased nuclear TDP-43.

Altogether, both Na-4-phenylbutyrate and piceatannol decreased a DSB DNA damage marker in our C9ALS MNs, even when variation across cell lines was considered. Our results suggest that small molecules targeting epigenetic modifiers, primarily HDAC inhibitors and SIRT1 activators, may have therapeutic potential, not only in the context of nucleocytoplasmic transport and cell viability, but in reducing DNA damage as well.

## 4. Discussion

ALS is a uniformly fatal disease caused by the degeneration of motor neurons [1]. A G_4_C_2_ repeat expansion in the *C9ORF72* gene is the most common genetic cause of ALS and FTD [4,5]. The repeat expansion mutation leads to the production of DPRs, three of which (PR, GR, and GA) are known to be more toxic than the other two (GP and PA) [17,29,66,76]. These DPRs lead to nucleolar dysfunction, impede the DNA damage repair response, and alter nucleocytoplasmic transport. Jovičić et al. first reported results from two genome-wide screens in yeast, identifying modifiers of PR toxicity that include karyopherins (importins and exportins), nuclear pore complex components, and enzymes involved in generating the Ran-GTP gradient that energetically drives nuclear transport [35]. Boeynaems et al. validated their findings in Drosophila, identifying 15 genes that “suppress” a PR-induced eye phenotype and 4 genes that “enhance” the PR eye phenotype [36]. Examples of suppressors of PR-toxicity are the karyopherins kap-alpha3 (KPNA3), transportin-1 (TNPO1), and XPO1, as well as RanGAP1, a regulator of the Ran-GTP cycle that drives nucleocytoplasmic transport. Subunits of the nuclear pore complex—NUP50, NUP107, and NUP155—were found to enhance PR toxicity.

These seminal studies, and others since then, have established that nucleocytoplasmic transport defects contribute to pathology in *C9ORF72* ALS/FTD. Despite this progress, the specific role of DPRs in non-classical nucleocytoplasmic transport pathways has not been systematically explored. Using a battery of nucleocytoplasmic transport biosensors, we have systematically defined the effects of toxic DPRs on all major nucleocytoplasmic transport pathways. We investigated how PR and two other toxic DPRs—GR and GA—affect nuclear export (Figure 3). Our results indicated that all three DPRs disrupted classical export mediated by XPO1, while none of the DPRs interfered with Aly/REF-mediated mRNA export. While mRNA processing is disrupted in *C9ORF72* ALS/FTD [59,60,61,62], our findings suggest this may not result from inhibition of the mRNA transport machinery directly. Rather, DPRs likely disrupt mRNA metabolism through the dysregulation of RNA binding proteins, such as TDP-43, NPM1, and hnRNPA3 that facilitate mRNA transport and are closely linked to ALS pathobiology [20,22,30,77,78].

A large family of karyopherins facilitate the import of cargo proteins via recognition of various classes of NLSs [43]. NLS domains contain essential amino acid residues that govern the binding affinity for specific importins and thus impact import activity [40,79]. The cNLS can be further classified as having one stretch of basic amino acids (monopartite) or two clusters of basic amino acids separated by a 9–12 amino acid linker (bipartite) [45,46]. The bipartite cNLS is utilized by TDP-43, whose mis-localization to the cytoplasm is a hallmark of ALS [70,73,74,75]. Our results in Figure 4 show that GA_50_ expression caused less nuclear accumulation of NCT-03 (our bipartite cNLS biosensor; Table 1) when compared with the extremely efficient XPO1 blocker LMB. Since XPO1-mediated export is disrupted by all three cytotoxic DPRs, lower nuclear accumulation of the NCT-3 biosensor, compared with LMB, suggests that GA may prevent import of cargo proteins that utilize the bipartite cNLS. In support of this, studies have shown that GA forms cytoplasmic aggregates and sequesters proteins [19,20,80]. Particularly, GA aggregates have been shown to prevent the nuclear import of TDP-43 [69].

In addition to cNLS, the PY-NLS and RS-NLS are well-characterized NLSs that are recognized by the karyopherins importin-β2 and transportin-SR, respectively [47,48]. While PY-NLS motifs are much more diverse in amino acid sequence, both PY-NLS and RS-NLS contain an overall positive charge and stretches of arginine-rich sequences like the cNLS. We found that PR_50_, GR_50_, and GA_50_ caused significantly less nuclear accumulation of our PY-NLS (NCT-07, NCT-08, NCT-09) and RS-NLS (NCT-13) biosensors when compared with LMB (Figure 4; with an exception for GR_50_, which instead increased nuclear accumulation of the biosensors compared to LMB). Furthermore, PR_50_, GR_50_, and GA_50_ caused a significant increase in nuclear localization of NCT-04, a biosensor that employs a non-classical NLS that is less arginine-rich than the cNLS, PY-NLS, and RS-NLS. There is evidence that DPRs disrupt import of cargo containing arginine-rich motifs by directly binding to importins and blocking the NLS binding site [71]. These findings suggest that DPRs pose an inhibitory effect on the import of cargo containing basic arginine-rich motifs as opposed to a non-classical NLS that is composed of less arginine, whose interaction with importin-α only partially overlaps the NLS binding site.

While our data supports recent findings that arginine-rich DPRs, primarily PR, directly bind to importins and impede nucleocytoplasmic transport [21,71]. Transport of proteins and mRNA is complex and our biosensors are simplified tools that allowed us to probe nucleocytoplasmic transport pathways. We developed the biosensors in accordance with the following criteria: (1) the NLS/NES must be sufficient and necessary for the transport of an endogenous protein; (2) the NLS/NES must be transferable, so as to direct the localization of an unrelated protein (such as GFP); and (3) the NLS/NES must directly and specifically interact with a karyopherin protein or complex. Unavoidable, but important limitations of this system that could affect biosensor transport efficiencies include: the NLS/NES location within the protein sequence; spurious protein–protein interactions between GFP and endogenous proteins; and the potential for post-translational modification of biosensors. Therefore, this approach to interrogate nucleocytoplasmic transport pathways may not be fully representative of the nucleocytoplasmic transport of endogenous proteins that utilize each transport pathway.

Genetic screens in yeast and *Drosophila* have provided evidence that alterations in the expression of nucleocytoplasmic transport machineries are linked to DPR toxicity [35,36]. However, more work must be carried out to translate these findings into therapies. It is possible that modulating the expression of multiple genes encoding nucleocytoplasmic transport machineries by pharmacological inhibition of epigenetic targets could be a viable therapeutic approach. In this study, we show that small molecules targeting epigenetic proteins, primarily HDAC inhibitors, may have therapeutic potential by restoring nucleocytoplasmic transport and increasing cell viability in PR_50_-expressing mammalian cells (Figure 5). Additionally, pan-HDAC inhibitor NA-4-phenylbutyrate and EZH1/EZH2 methyltransferase inhibitor UNC 1999 reduced nuclear localization of PR_50_ in mammalian cells (Figure S6). However, it is important to note that the U-2 OS cells do not harbor the *C9ORF72* expansion, and the PR expression was driven by a strong constitutive CMV promoter. We show that “synthetic” PR_50_ localizes to the nucleus and nucleolus, consistent with previous studies using DPR expression plasmids (Figure S4) [25]. However, the localization of synthetic DPRs is dependent on repeat length and cellular environment. In non-neuronal cells, both PR and GR formed more cytoplasmic inclusions when compared with neuronal cell lines [25,76]. Thus, synthetic DPRs may differ from endogenous DPRs in their localization and abundance.

While we investigated over 2000 compounds, we were not able to include the selective inhibitor of nuclear export (SINE) compounds KPT-335 or KPT-350, as they were not commercially available when we began the screening campaign. The SINE compounds inhibit nuclear export by targeting exportin-1. The compounds extend survival in neuronal ALS/FTD models and reduce motor symptoms in a rat ALS model [81], while neither the SINE compounds nor LMB increased nuclear TDP-43 levels [81].

While PR_50_ had preponderate effects on nucleocytoplasmic transport, other mechanisms likely contribute to its potently cytotoxic effects. For example, a two hour inhibition of nucleocytoplasmic transport using LMB did not impact overall cell viability. Therefore, the cytotoxic effects observed after 24 h of PR expression could result from multiple mechanisms, such as inhibition of DNA damage repair, as we have previously reported [30]. Therapeutic strategies to mitigate DPR toxicity may, therefore, require restoration of nucleocytoplasmic transport efficiency and other phenotypes, such as increased rates of DNA damage and reduced repair efficiencies.

We found that motor neurons are morphologically incompatible with our nucleocytoplasmic transport assay. Therefore, we tested whether hit compounds are capable of rescuing an alternative disease-relevant cellular phenotype in C9ALS/FTD MNs. We previously found increased DNA damage to be a robust and well-established phenotype that can be easily quantified by γH2AX immunoreactivity [30]. We prioritized hit compounds according to their ability to increase cell viability of U-2 OS cells expressing PR_50_ and reduced target redundancy (i.e., avoiding the use of several EZH1/EZH2 inhibitors). Several HDAC inhibitors have been pursued as therapy for neurodegenerative disorders associated with the loss of acetylation homeostasis [10,82,83,84,85,86]. For example, Na-4-phenylbutyrate, a brain-penetrant pan HDAC inhibitor, increased cell viability in PR-expressing U-2 OS cells [87], prolonged the survival and regulated the expression of anti-apoptotic genes in transgenic ALS mice [88], and has been investigated as a therapeutic in conjunction with taurursodial for ALS in a recent clinical trial (NCT03127514) [89]. Our findings show that Na-4-phenylbutyrate rescues DPR-induced nucleocytoplasmic transport deficiencies and alleviates increased DNA DSB frequency in patient iPSC motor neurons (Figure 5 and Figure 6). Several other studies show HDAC inhibition promotes survival or alleviates neurodegenerative pathomechanisms. The inhibition of HDAC6, for example, restores TDP-43 mis-localization and fragmentation in human motor neurons with mutations in *TARDBP* [72], and restores mitochondrial function and axonal transport defects in patient-derived motor neurons [90]. Here, we found that CAY10603, a potent HDAC6 inhibitor, decreases DNA DSBs in patient-derived MNs (Figure 6). However, neither CAY10603 nor any of the tested hit compounds restored TDP-43 mis-localization in C9ALS-3 MNs (Figure S9).

To address variability across iPSC lines, we used well-characterized iPSC lines, created in the Zeier laboratory, as well as available lines from the Cedars Sanai iPSC core facility. To minimize the potential confounding effects of background genetic variability, we used genetically corrected isogenic lines, in which the *C9ORF72* expansion was excised by CRISPR/Cas9 mediated genome editing. Despite these efforts to control for inter-individual variability, we saw variable effects of compound treatments on C9ALS MNs from different iPSC lines. While this is an inherent limitation of human-based model systems, an advantage is the potential for developing personalized therapeutic approaches for a subset of patients with the most potential for therapeutic effect.

The power of using a phenotypic screen is that the knowledge of the disease mechanism is not entirely needed to identify compounds capable of reversing said phenotype. However, a weakness of the strategy is that the mechanism of phenotypic rescue is difficult to ascertain without further experimentation. We have previously shown that epigenetic compounds, particularly HDAC inhibitors and bromodomain inhibitors, alter the expression of the endogenous *C9ORF72* locus [91]. Specifically, PCI-24781 (pan HDAC inhibitor), RG 2833 (HDAC 1 and 3 inhibitor), and CI-994 (selective class I HDAC inhibitor) were among the top 20 hits from said screen. However, these compounds did not increase cell viability in our PR-expressing U-2 OS cells (Figure 5D), so they were not investigated further in our C9ALS MNs. It is possible that altering C9ORF72 expression is not required for increasing cell survival and restoring disrupted NCT, but this was not examined directly in cells with the endogenous *C9ORF72* HRE. It is also possible that epigenetic modulation via small molecules treatment increases the expression of essential NCT proteins. Nuclear pore proteins and other components of NCT machinery, such as RanGAP1, are mis-localized and sequestered in the cytoplasm [69,92]. Thus, epigenetic modulation may facilitate nuclear transport and restore the Ran gradient. Future studies will be needed to determine how epigenetic small molecules identified in this study confer their therapeutic effects. Evaluation of gene expression changes, combinatorial synergistic effects, and effects on alternative phenotypes in animal models will be needed to enable clinical trials. Nevertheless, we have identified and validated at least five drug-like small molecules with therapeutic potential against C9ALS/FTD pathology.

## 5. Conclusions

Here, we show that PR_50_, GR_50_, and GA_50_ contribute to the disruption of classical nuclear export, classical nuclear import, and non-classical nucleocytoplasmic transport pathways. By conducting a cell-based phenotypic screen, we identified compounds with the ability to alleviate PR_50_-induced toxicity. In addition to identifying a compound that is already in clinical trials for the treatment of ALS (Na-4-phenylbutyrate), our screen also identified other HDAC inhibitors, EZH1/EZH2 inhibitors, HAT activators, and SIRT1 activators that improved cell viability and restored nucleocytoplasmic transport in PR_50_-expressing cells. Several of these small molecules decreased DNA damage markers in C9ALS/FTD patient iPSC-derived motor neurons. In summary, we provide evidence that small molecules targeting epigenetic proteins are capable of modifying the toxicity induced by ALS-related DPRs.

## Figures and Tables

**Table 1:** NLS and NES classes and sequences of each nucleocytoplasmic transport biosensor.

Biosensor	NLS Type	NLS Sequence	Importin Recognized	NES Type	NES Sequence	Exportin Recognized
NCT-C	cNLS	PKKKRKV	KNPA1-KPNB1	cNES	KEVDQLRLERLQIDEQL	XPO1
NCT-01	cNLS R5A mutant	PKKKAKV	KNPA1-KPNB1	cNES	KEVDQLRLERLQIDEQL	XPO1
NCT-02	cNLS K6A mutant	PKKKRAVE	KNPA1-KPNB1	cNES	KEVDQLRLERLQIDEQL	XPO1
NCT-03	cNLS (bipartite)	KRPAATKKAGQAKKKK	KNPA1-KPNB1	cNES	KEVDQLRLERLQIDEQL	XPO1
NCT-04	Non-classical NLS	GKISKHWTG	KNPA1-KPNB1	cNES	KEVDQLRLERLQIDEQL	XPO1
NCT-05	IB1-NLS	RRKKKEYVK	KNPB1	cNES	KEVDQLRLERLQIDEQL	XPO1
NCT-06	IB1-NLS	RKKRRQRRR	KNPB1	cNES	KEVDQLRLERLQIDEQL	XPO1
NCT-07	PY-NLS (basic, M9)	FGNYNNQSSNFGPMKGGNFGGRSSGPY	KNPB2	cNES	KEVDQLRLERLQIDEQL	XPO1
NCT-08	PY-NLS (hydrophobic)	YGDYSNQQSGYGKVSRRGGHQNSYKPY	KNPB2	cNES	KEVDQLRLERLQIDEQL	XPO1
NCT-09	PY-NLS (atypical)	GPGKMDSRGEHRQDRR-ERPY	KNPB2	cNES	KEVDQLRLERLQIDEQL	XPO1
NCT-10	I4-NLS	GKVSKRKAV	IPO4	cNES	KEVDQLRLERLQIDEQL	XPO1
NCT-11	I5-NLS	HTPQRVLPLKKPPMKSLRKKGSGKILTPAKKSFL	IPO5	cNES	KEVDQLRLERLQIDEQL	XPO1
NCT-13	RS-NLS (RD mimic)	RDPSYG(RD)8NDRDRDYSPRRDRGSPRYSPRHDRDRDRT	TNPO3	cNES	KEVDQLRLERLQIDEQL	XPO1
NCT-14	Homeodomain-NLS	RKLQRNRTSFTQEQIEALEKEFERTHYPDVFARERLAAKIDLPEARIQVWFSNRRAKWRREE	IPO13	cNES	KEVDQLRLERLQIDEQL	XPO1
NCT-15	cNLS (monopartite)	PKKKRKV	KNPA1-KPNB1	cNES	KEVDQLRLERLQIDEQL	XPO1
NCT-A	cNLS	PKKKRKV	KNPA1-KPNB1	NXF1-NXT1 mRNA	APPAQPPSQPQQHYSEGELEEDEDSDDA	ALYREF Adapter

## Data Availability

The datasets supporting the conclusions of this article are included within the article and its supplementary materials.

## Supplementary Materials

The following are available online at https://www.mdpi.com/article/10.3390/brainsci11111543/s1: Figure S1. Confirmation of motor neuron markers in iPSC-derived motor neurons (Related to Figure 6). File format: PDF (.pdf). Confocal images of iPSC-derived motor neurons used in the RT qPCR experiment. Neurons were stained for neuronal marker Tuj1 and motor neuron-specific marker Isl1 to confirm proper differentiation. Scale bar = 60 µm. Figure S2. Schematic of nucleocytoplasmic transport biosensor plasmids (Related to Figure 1). File format: PDF (.pdf). Feature map of the nucleocytoplasmic biosensor plasmids. Linear representation of the fusion protein, green fluorescent protein (GFP) fused with nuclear localization signal (NLS) and nuclear export signal (NES). Plasmid map generated in Snapgene. Figure S3. Representative images of NCT biosensors treated with LMB (Related to Figure 1). File format: PDF (.pdf). Confocal images of U-2 OS NCT biosensor cells used in the LMB test experiment (Figure 1C). Cells were treated with 2 ng/mL LMB for 2 h vs treatment with vehicle (DMSO). Scale bar = 50 µm. Figure S4. ImageJ analysis of DPR localization in NCT-C cells (Related to Figure 3). File format: PDF (.pdf). Confocal images of NCT-C cells after 24 h exposure to PR50, GR50, or GA50 expression plasmids. The DPR channel (top images) were background corrected and the nuclei (bottom images) were traced and overlayed onto the DPR channel. All analyses were carried out using the Intensity Ratio Nuclei Cytoplasm Tool in ImageJ (Intensity Ratio Nuclei Cytoplasm Tool, RRID:SCR_018573 ). Figure S5. Assay robustness of all plates utilized in primary screen (Related to Figure 5). File format: PDF (.pdf). Bar graph showing the calculated Z-factor between DMSO and LMC control wells across all plates analyzed for the high-content primary screen. A Z-factor of at least 0.5 (red dotted line) indicates an excellent assay. Figure S6. Translocation of PR in NCT-C cells after treatment with hit compounds (Related to Figure 5). File format: PDF (.pdf). A scatter plot of the nuclear–cytoplasm difference of PR intensity in NCT-C cells treated with hit compounds from the confirmatory screen in Figure 5C,D. Cells were either treated with vehicle (DMSO), untreated and expressing PR50 (PR), or treated with compound at 3 µM for 24 h in the presence of PR50. Each condition was repeated 4 times. Data were normalized to DMSO (0 = no nuclear PR) and PR (1 = predominantly nuclear PR). Each data point represents a single well. A one-way ANOVA with Dunnett’s post-hoc test was performed, comparing each condition to PR. Asterisks indicate significantly less nuclear PR when compared with the PR-only condition. Figure S7. Average nuclear γH2AX fluorescence intensity in C9ALS iPSC MNs and their isogenic controls (related to Figure 6). File format: PDF (.pdf). (A) Quantification of average γH2AX nuclear fluorescence intensity in each C9ALS MN (dark grey; + vehicle) compared to their corresponding isogenic controls (light grey; + vehicle). 1 h of 200 μM etoposide treatment was performed in each C9ALS MN (dark green) and their isogenic controls (light green) as a positive control to induce DNA DSBs. n = 3 biological replicates; 3 fields imaged and analyzed per replicate. Each dot represents a single cell. One-way ANOVA with multiple comparisons using Sidak’s correction; F (11, 13232) = 907.6; **** *p* < 0.0001; * *p* < 0.05. (B) Representative confocal images for data represented in (A) showing DAPI nuclei staining and γH2AX (orange). Scale bar = 40 μm. (C) Magnified confocal images of C9ALS-1 and Isogenic-1 MNs treated with either vehicle (DMSO) or 200 μM etoposide. The magnified images show nuclei (DAPI) with or without accumulation of γH2AX (orange). Scale Bar = 20 μm. Figure S8. Average nuclear γH2AX fluorescence intensity in C9ALS-3 MNs treated with several doses of each small molecule (related to Figure 6). File format: PDF (.pdf). Quantification of average nuclear γH2AX fluorescence intensity in Isogenic-3 and C9ALS-3 MNs, as well as C9ALS-3 MNs treated with three different doses of each small molecule tested (1 X IC50, 10 X IC50, and 100 X IC50). Each data point represents a single cell. Student’s T-test was performed comparing each condition with the C9-3 condition. Asterisks denote significance relative to C9-3. ** *p* < 0.01; * *p* < 0.05. Semi-automated image analysis was done using confocal imaging and ImageJ. Statistical analysis performed in Graphpad Prism. Figure S9. Average nuclear TDP-43 fluorescence intensity in C9ALS MNs treated with epigenetic small molecules (related to Figure 6). File format: PDF (.pdf). Quantification of average nuclear TDP-43 fluorescence intensity in Isogenic and C9ALS MNs, as well as C9ALS MNs treated with each hit small molecule. Each data point represents a single cell. Student’s T-test was performed comparing each C9ALS MN with its corresponding isogenic control. Table S1: Antibody applications and dilutions. File format: PDF (.pdf). Table S2. *p*-values for statistical tests (Related to Figure 3 and Figure 4). File format: Microsoft Excel Worksheet (.xlsx). Table S3. Information on compound libraries used (Related to Figure 5). File format: PDF (.pdf). Table S4. Results from cell-based phenotypic primary screen (Related to Figure 5). File format: Microsoft Excel Worksheet (.xlsx). Table S5: *p*-values for statistical tests (Related to Figure 6). File format: Microsoft Excel Worksheet (.xlsx).
