# Supplementary material for: Epigenetic Small Molecules Rescue Nucleocytoplasmic Transport and DNA Damage Phenotypes in C9ORF72 ALS/FTD
Source: Brain Sci. 2021 Nov 20;11(11):1543. doi: 10.3390/brainsci11111543 (PMC8616043; doi:10.3390/brainsci11111543)
Supplement: Supplementary file 1 [file brainsci-11-01543-s001.zip › Figure S4.pdf]

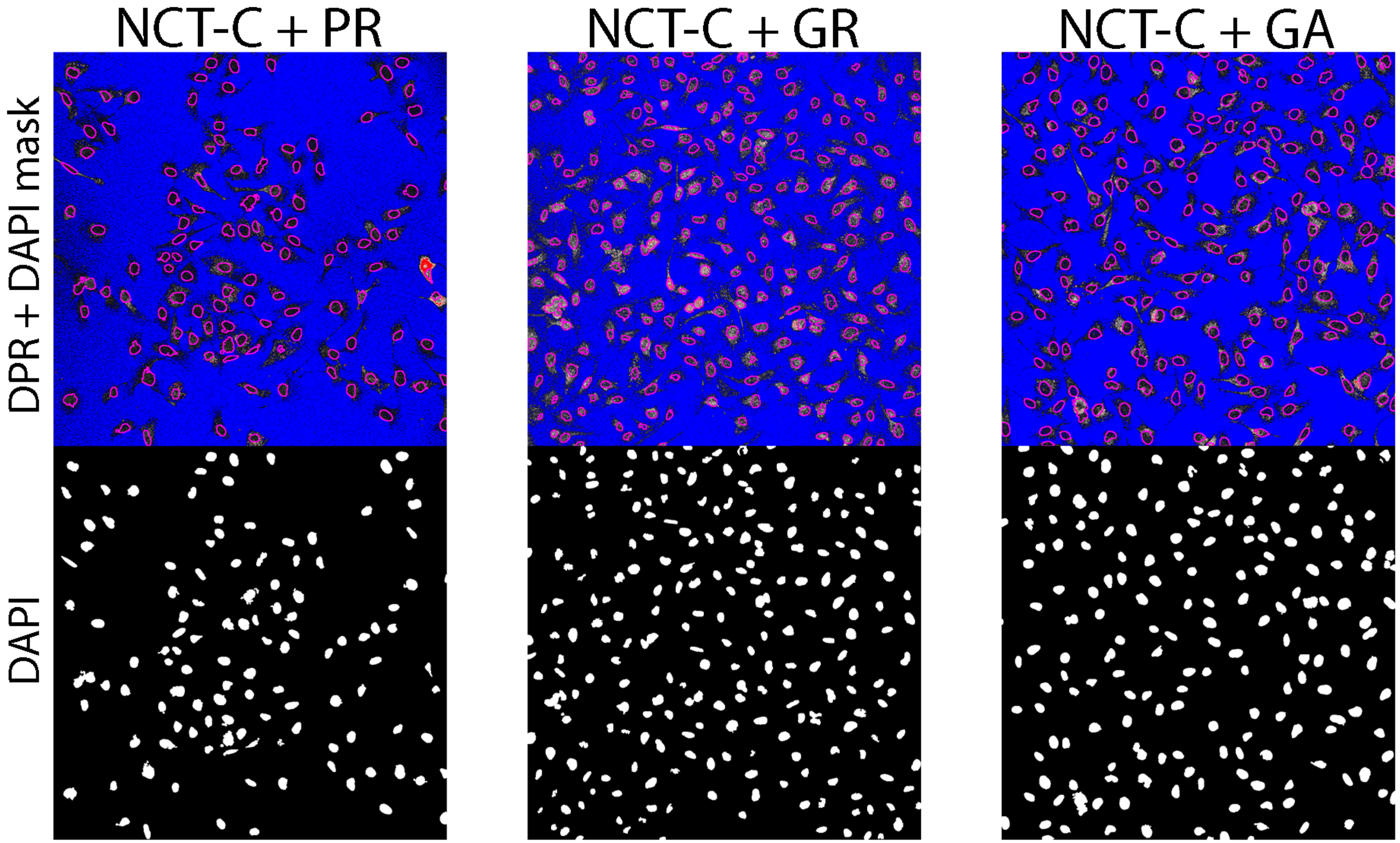

Image	av. nuclei intensity	av. cytoplasm intensity	t. nuclei intensity	t. cytoplasm intensity
NCT_C_PR	13.445	10.3021	410760.449	976026.758
NCT_C_GR	10.5866	6.4637	487623.47	712430.68
NCT_C_GA	13.6998	17.0434	682618.094	1535388.19
