# Supplementary material for: Epigenetic Small Molecules Rescue Nucleocytoplasmic Transport and DNA Damage Phenotypes in C9ORF72 ALS/FTD
Source: Brain Sci. 2021 Nov 20;11(11):1543. doi: 10.3390/brainsci11111543 (PMC8616043; doi:10.3390/brainsci11111543)
Supplement: Supplementary file 1 [file brainsci-11-01543-s001.zip › Table S1.pdf]

Table S1. Antibody applications and dilutions

Antibody	Supplier	Catalog #	Dilution	Method
anti-PR	ProteinTech	23979-1-AP	1:250	IF
anti-GR	ProteinTech	23978-1-AP	1:100	IF
anti-GA	ProteinTech	24492-1-AP	1:100	IF
anti-yH2AX	Abcam	ab11174	1:1000	IF
anti-Tuj1	Abcam	ab78078	1:1000	IF
anti-ISL1	Abcam	ab20670	1:500	IF
anti-TDP-43	ProteinTech	10782-2-AP	1:250	IF
