# Supplementary material for: Epigenetic Small Molecules Rescue Nucleocytoplasmic Transport and DNA Damage Phenotypes in C9ORF72 ALS/FTD
Source: Brain Sci. 2021 Nov 20;11(11):1543. doi: 10.3390/brainsci11111543 (PMC8616043; doi:10.3390/brainsci11111543)
Supplement: Supplementary file 1 [file brainsci-11-01543-s001.zip › Table S3.pdf]

Table S3. Compound Libraries

Name	Supplier	Catalog #	# of compounds
LOPAC®1280 - Small Scale	Sigma-Aldrich	LO4200-1EA	1280
SCREEN-WELL® FDA approved drug library	Enzo	BML-2841	800
SCREEN-WELL® Natural Product library	Enzo	BML-2865	502
CTI Epigenetic Compound Library	N/A	N/A	160
		**Total Compounds**	**2742**
