# Supplementary figures and images for: Epigenetic Small Molecules Rescue Nucleocytoplasmic Transport and DNA Damage Phenotypes in C9ORF72 ALS/FTD
Source: Brain Sci. 2021 Nov 20;11(11):1543. doi: 10.3390/brainsci11111543 (PMC8616043; doi:10.3390/brainsci11111543)

**Figure S1. Confirmation of motor neuron markers in iPSC-derived motor neurons**

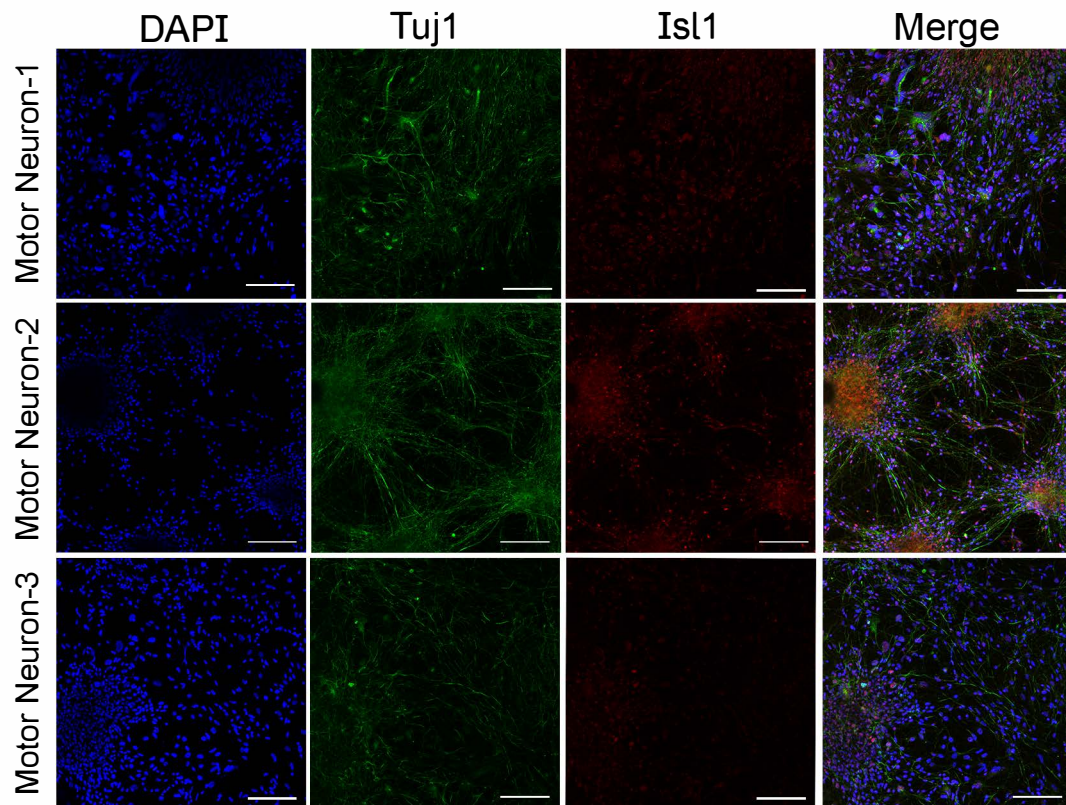

Supplement: Supplementary file 1 [file brainsci-11-01543-s001.zip › Figure S1.pdf]

NLS

GFP

NES

Created with SnapGene®

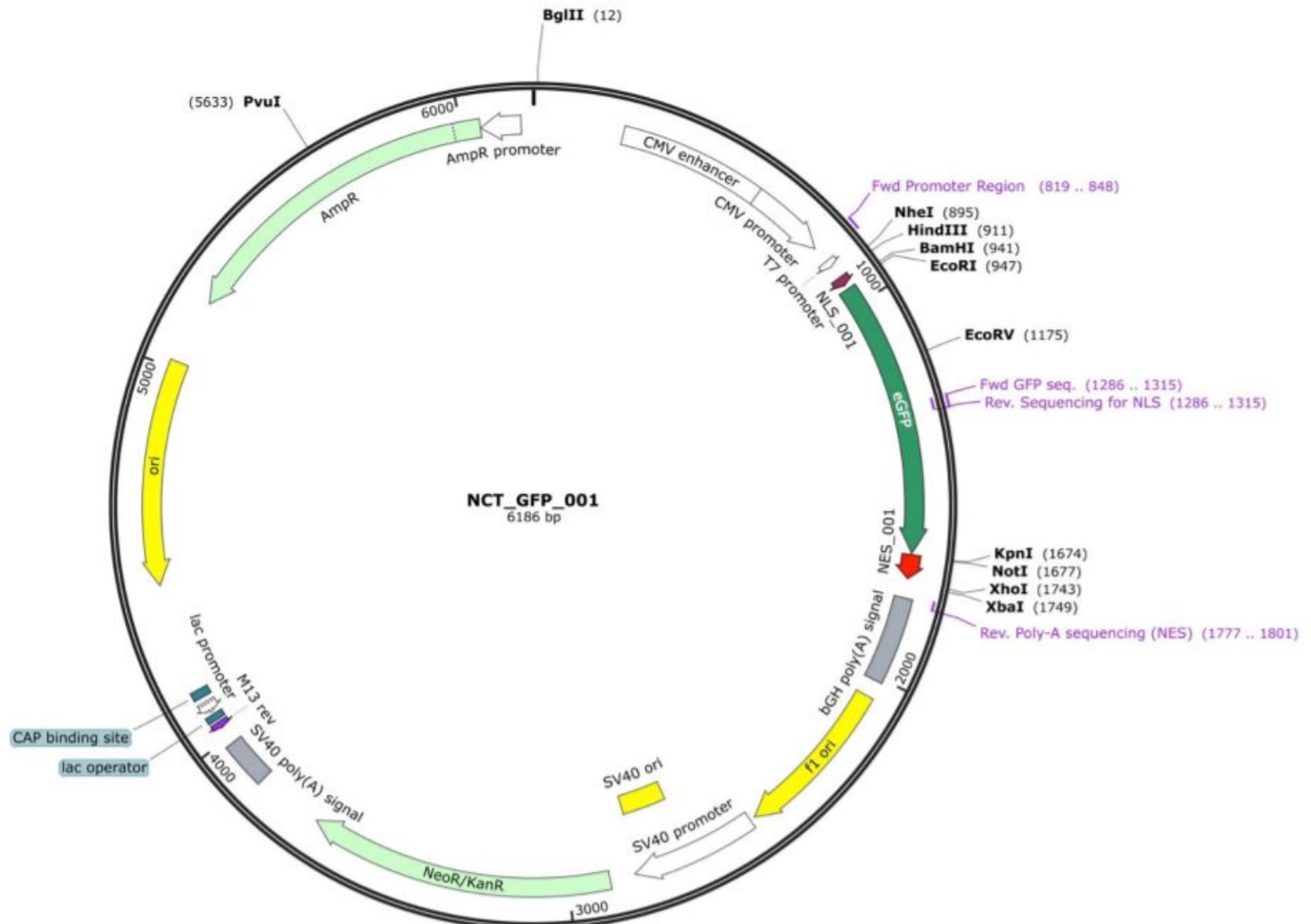

Supplement: Supplementary file 1 [file brainsci-11-01543-s001.zip › Figure S2.pdf]

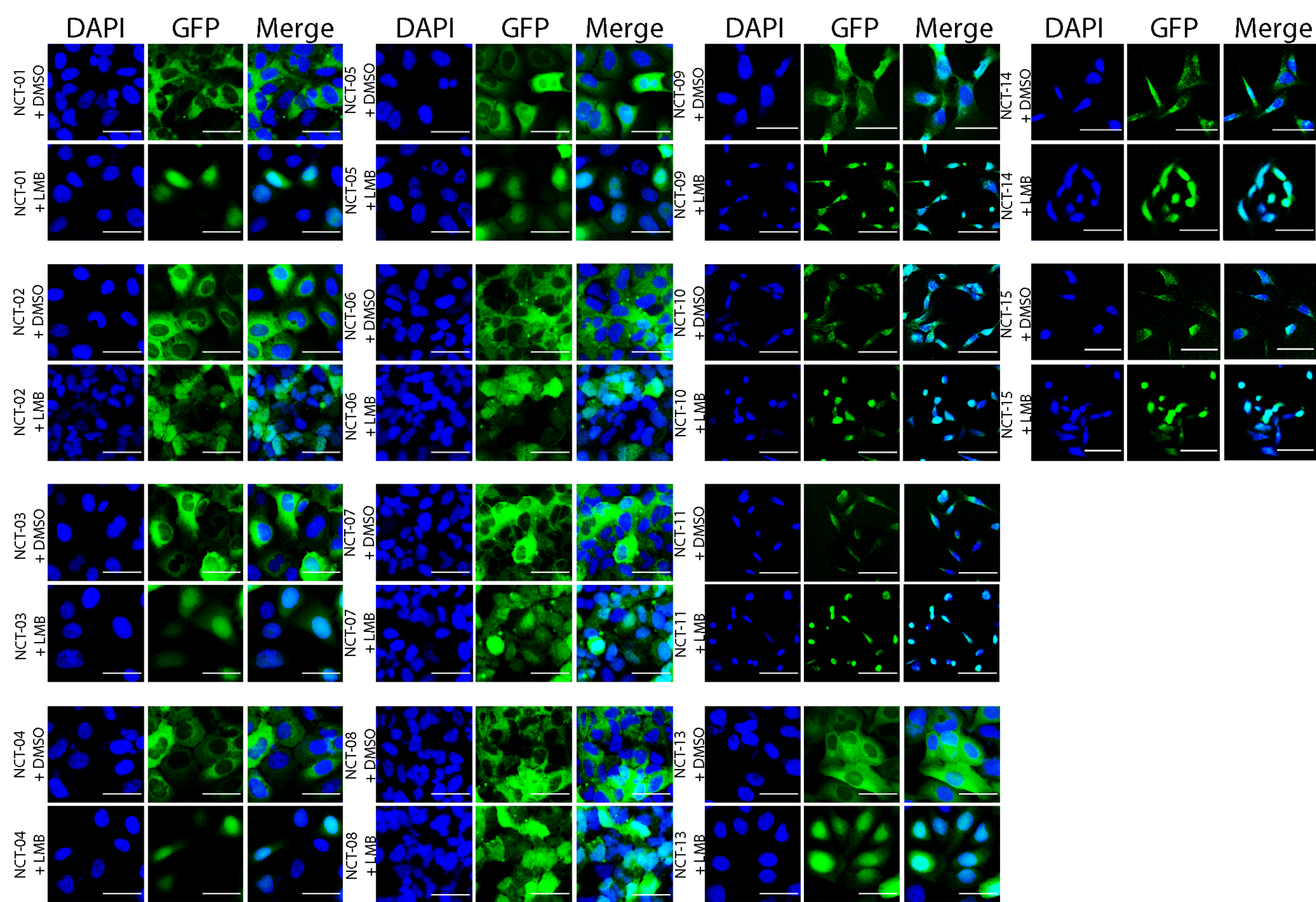

Supplement: Supplementary file 1 [file brainsci-11-01543-s001.zip › Figure S3.pdf]

# Assay Robustness

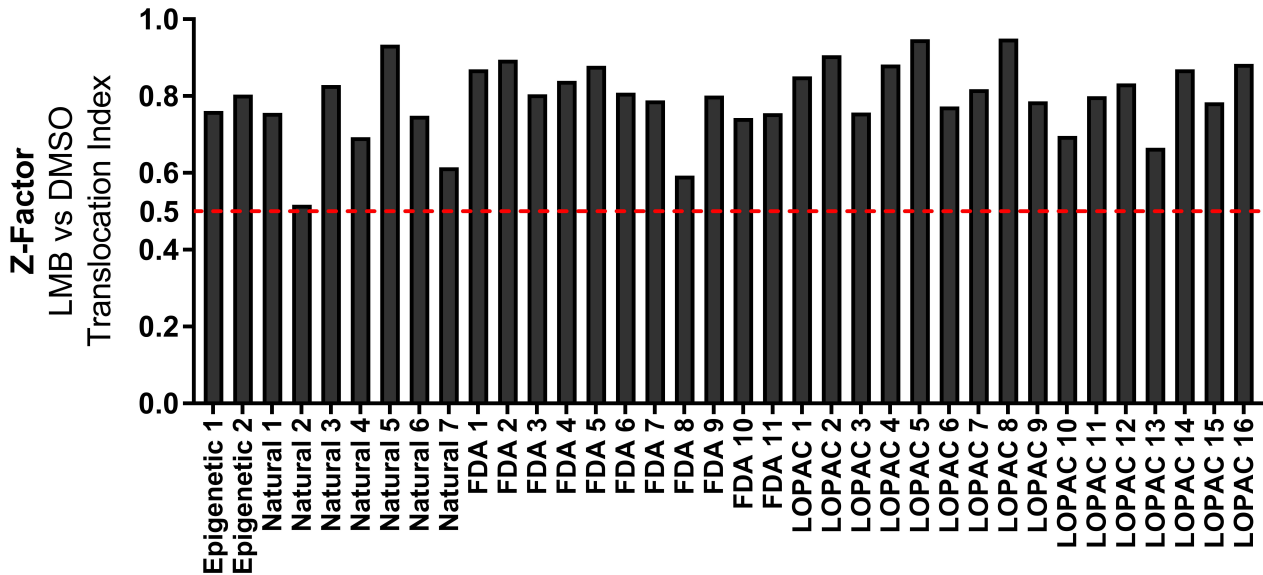

Supplement: Supplementary file 1 [file brainsci-11-01543-s001.zip › Figure S5.pdf]

Translocation Index of PR  
Normalized to DMSO (0)  
and PR (1)

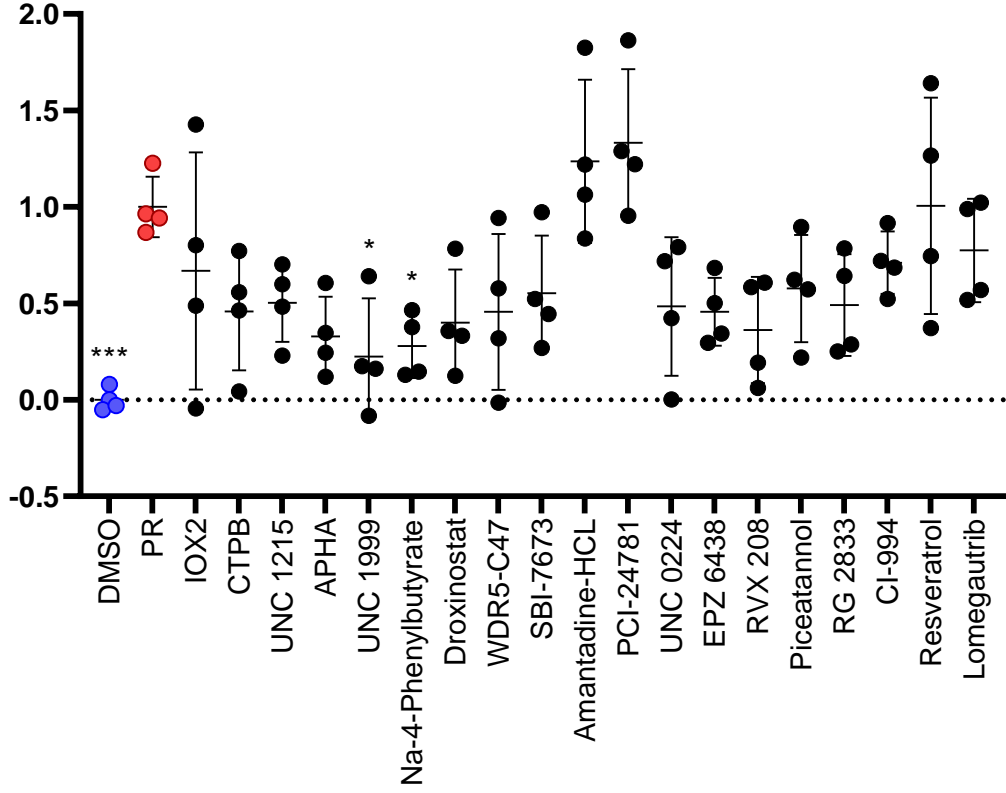

Supplement: Supplementary file 1 [file brainsci-11-01543-s001.zip › Figure S6.pdf]

**A**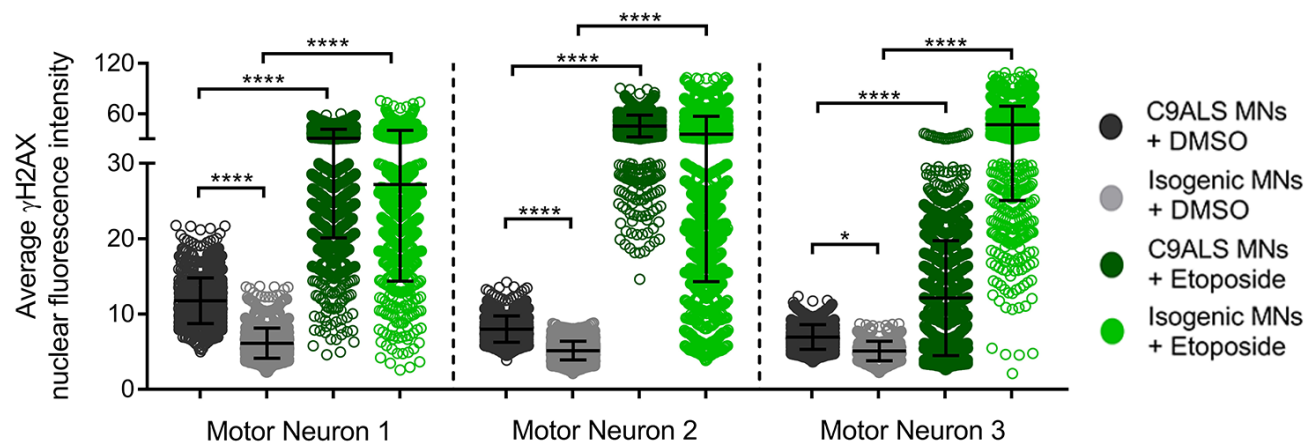**B**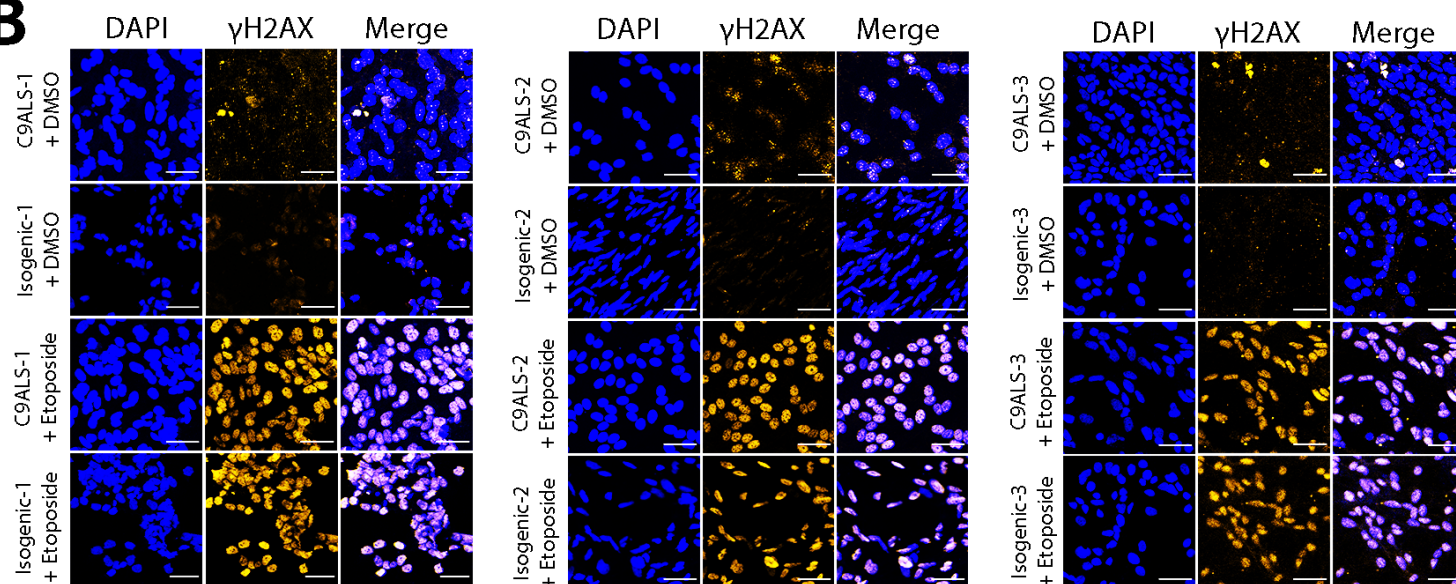**C**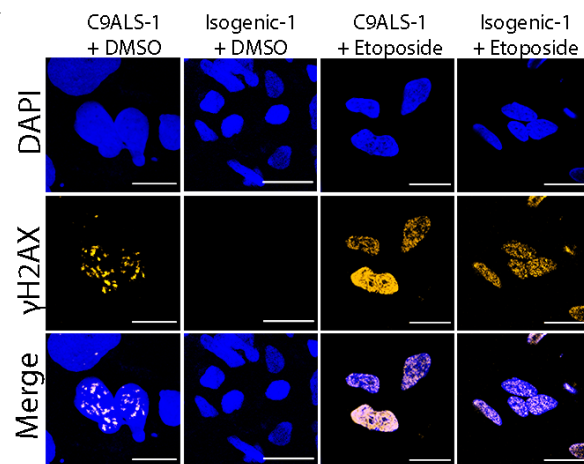

Supplement: Supplementary file 1 [file brainsci-11-01543-s001.zip › Figure S7.pdf]

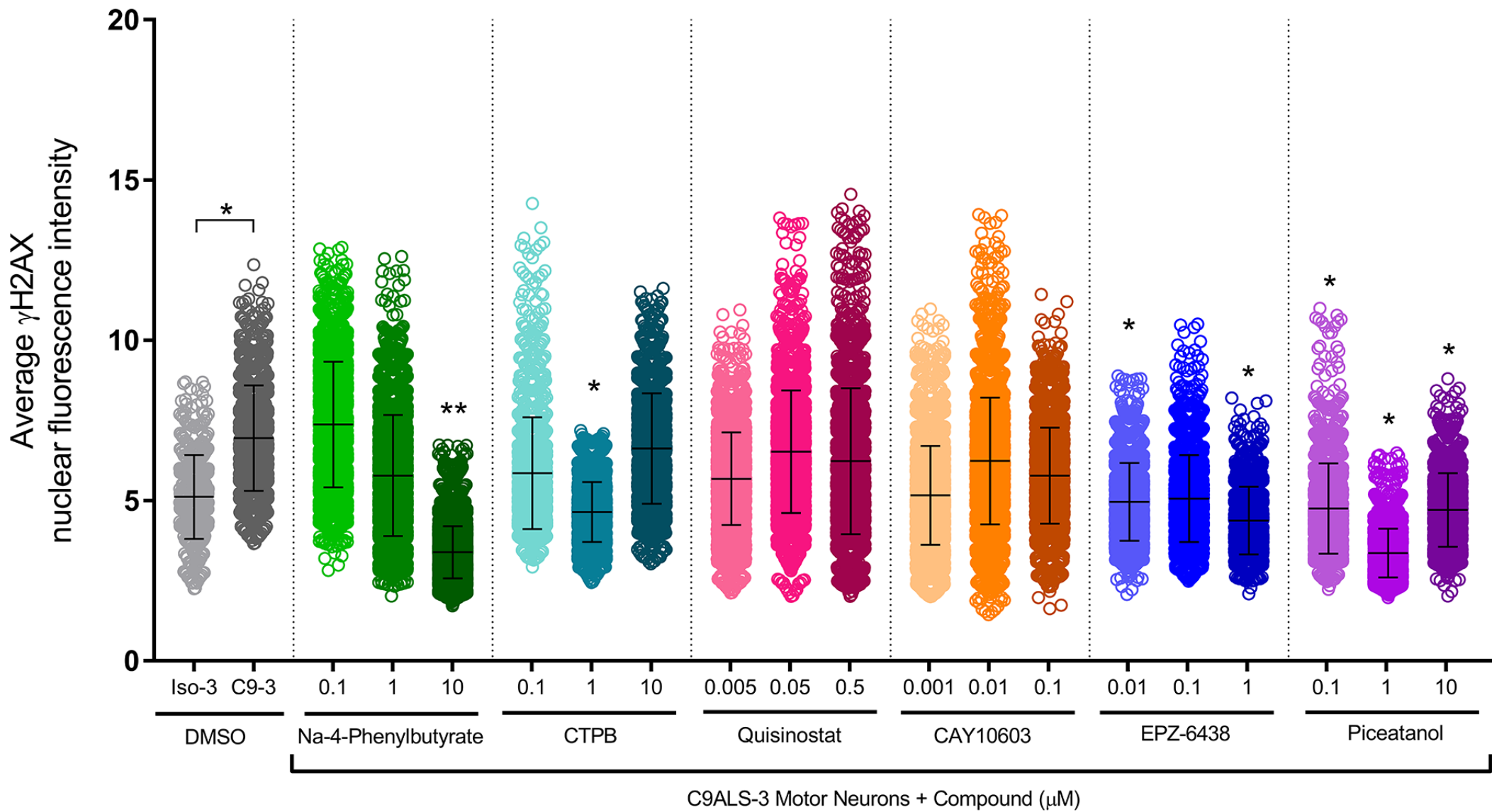

Supplement: Supplementary file 1 [file brainsci-11-01543-s001.zip › Figure S8.pdf]

**A**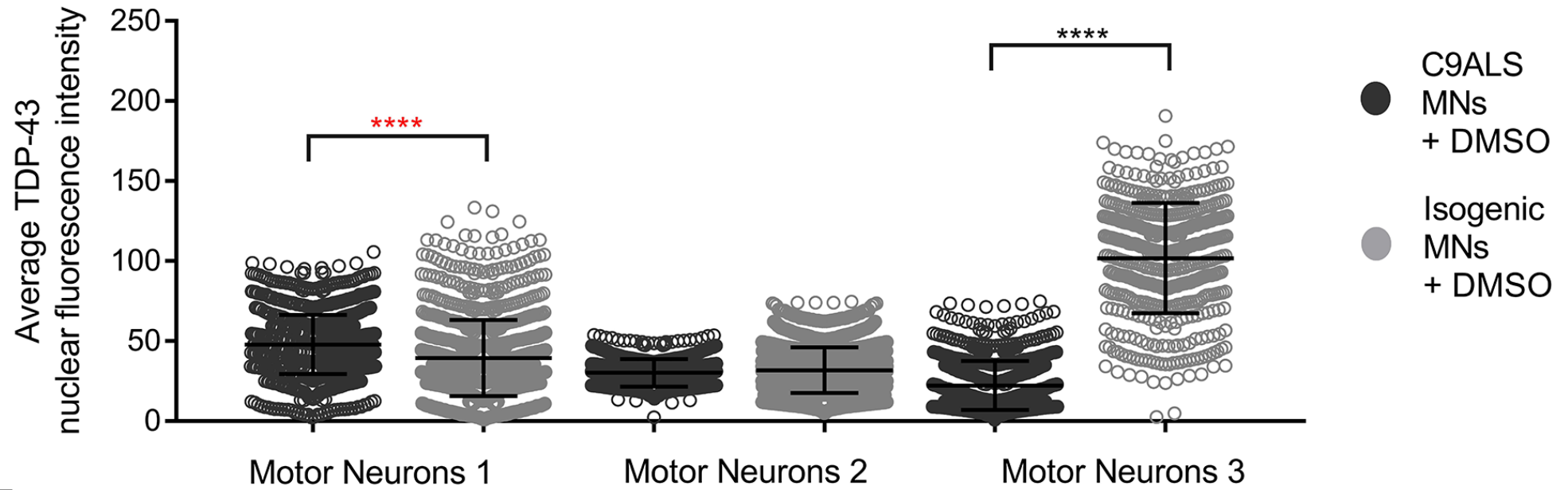**B**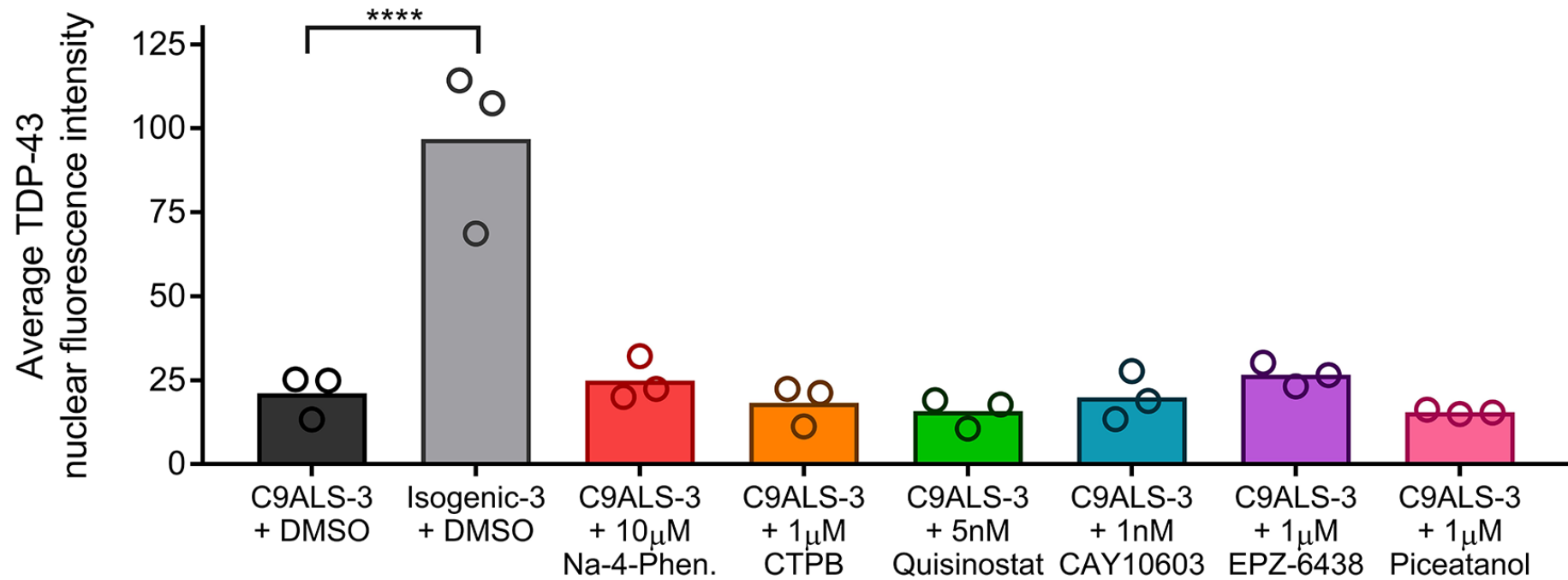

Supplement: Supplementary file 1 [file brainsci-11-01543-s001.zip › Figure S9.pdf]
